# Preclinical assessment of broadly neutralizing HIV-1 antibody BNT351 with optimized pharmacokinetics and potent antiviral activity

**DOI:** 10.1016/j.isci.2026.116022

**Published:** 2026-06-11

**Authors:** Sven Kratochvil, Maximilian Kullmann, Henning Gruell, Sophie Sayettat, Chia-Hung Tsai, Natasa Vukovic, Christine Janaitis, Claudia Lindemann, Sandra Praßl, Ricarda Stumpf, Jacqueline Knüfer, Felix Tolksdorf, Uğur Şahin, Johannes Nelke, Alexandra Malz, Philipp Schommers, Sinethemba Bhebhe, Nonhlanhla Mkhize, Penny Moore, Michael S. Seaman, Florian Klein, Valentin Le Douce

**Affiliations:** 1 BioNTech SE, Mainz, Germany; 2 Institute of Virology, Faculty of Medicine and University Hospital Cologne, University of Cologne, Cologne, Germany; 3 BioNTech US Inc., Cambridge, MA, USA; 4 BioNTech UK Ltd, London, UK; 5 HI-TRON - Helmholtz Institute for Translational Oncology Mainz by DKFZ, Mainz, Germany; 6 TRON gGmbH - Translational Oncology at the University Medical Center of the Johannes Gutenberg University, Mainz, Germany; 7 Department I of Internal Medicine, Faculty of Medicine and University Hospital Cologne, University of Cologne, Cologne, Germany; 8 German Center for Infection Research (DZIF), Partner Site Bonn-Cologne, Cologne, Germany; 9 Center for Molecular Medicine Cologne (CMMC), University of Cologne, Cologne, Germany; 10 SA MRC Antibody Immunity Research Unit, School of Pathology, Faculty of Health Sciences, University of the Witwatersrand, Johannesburg, South Africa; 11 National Institute for Communicable Diseases of the National Health Laboratory Service, Johannesburg, South Africa; 12 Wits Infectious Diseases and Oncology Research Institute, Faculty of Health Sciences, University of the Witwatersrand, Johannesburg, South Africa; 13 Centre for the AIDS Programme of Research in South Africa (CAPRISA), University of Kwa-Zulu Natal, Durban, South Africa; 14 Center for Virology and Vaccine Research, Beth Israel Deaconess Medical Center, Harvard Medical School, Boston, MA, USA

**Keywords:** Immunology, virology, biotechnology, antibody therapy, HIV-1, pharmacokinetics, bNAb

## Abstract

Broadly neutralizing antibody (bNAb) 1-18 is a promising tool for future clinical strategies against HIV-1 infection. To enhance 1-18’s clinical potential, we introduced half-life-extending LS mutations and evaluated the resulting investigational bNAb candidate, BNT351. LS mutations increased the affinity of BNT351 to human neonatal Fc receptor by 20-fold, resulting in a long half-life of 10–14 days in Tg32 mice and 18 days in non-human primates, with a predicted human half-life of ∼50 days. BNT351 retained 1-18’s exceptional neutralization potency and breadth against a 119 multiclade panel, and neutralized a pseudovirus panel of circulating HIV-1 clade C strains with high potency. BNT351 fully suppressed viremia in HIV-1-infected humanized CD34^+^ NSG mice without eliciting resistant viral variants. Additionally, we observed no off-target binding of BNT351 to a panel of ∼6,500 human proteins, and no developability concerns. This favorable preclinical evaluation supported initiation of a phase 1 clinical trial with BNT351 (NCT07392372).

## Highlights

• BNT351 is half-life-optimized HIV bNAb 1-18, showing favorable PK properties in NHPs
• BNT351 exhibits high neutralization potency against clade C circulating strains
• BNT351 fully suppresses viremia in HIV-1__YU2__-infected humanized mice
• No off-target binding to human proteins and no developability concerns detected

## Graphical abstract

**Figure:**
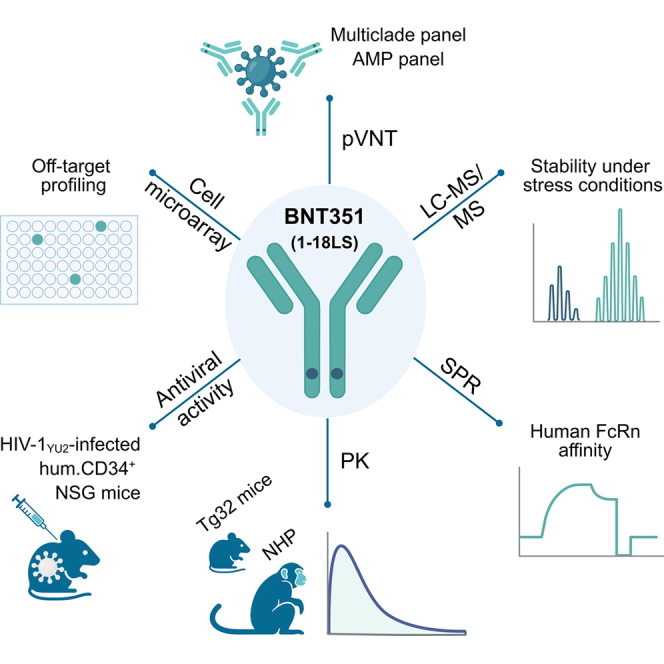

## Introduction

Broadly neutralizing antibodies (bNAbs) provide a promising therapeutic strategy to combat HIV-1. These highly potent antibodies target conserved regions on the viral envelope and can neutralize the majority of circulating HIV-1 strains.^1^ However, early bNAb clinical trials showed only transient antiviral activity of individual antibodies, mostly due to viral escape mutations, which could be improved by combining multiple bNAbs.^2^ Compelling preclinical activity suggests that bNAb 1-18, isolated from a clade B-infected long-term non-progressor, could become the centerpiece of rationally designed bNAb therapeutic cocktails for broadened coverage and reduced viral escape risk. 1-18 targets the CD4 binding site (CD4bs) on the HIV-1 envelope protein (Env) and has shown exceptional neutralization breadth (97%) and potency (GeoMean half maximal inhibitory concentration [IC_50_] = 0.048 μg/mL) combined with restricted viral escape.^3^ Monotherapy with 1-18 maintained viral suppression in HIV-1-infected humanized mice without raising resistant viral variants, whereas escape variants were seen for other CD4bs bNAbs in the same model.^3^

Recent clinical trials have shown that viral rebound can occur when bNAb serum concentrations fall below 10 μg/mL.^2^ Similarly, maintaining bNAb titers above a threshold of 200×IC_80_ has been proposed for protection efficacy.^4^ This highlights the need for potent and long-acting bNAbs for less frequent dosing. Thus, before moving 1-18 to clinical testing for HIV therapy, we aimed to optimize its half-life by introducing half-life-extending LS mutations in the Fc region, giving rise to BNT351 bNAb. LS mutations (M428L/N434S; Xtend) have been designed to extend IgG half-life and have been validated for human use with several monoclonal antibodies either in clinical development or marketed,^5^^,^^6^ including HIV bNAbs.^7^ LS-modified IgG variants show several fold higher affinity for neonatal Fc receptor (FcRn) and, subsequently, higher cellular recycling and prolonged serum half-life *in vivo.*^8^

The aim of this study was to provide an extended preclinical assessment of BNT351 prior to first-in-human testing. To confirm the effect of LS mutations, we evaluated target and human Fc receptor (hFcR) binding *in vitro*, and assessed BNT351 pharmacokinetic (PK) profiles in Tg32 mice and cynomolgus macaques. We further expanded available 1-18 data to include off-target profiling and neutralization activity against breakthrough viruses from the Antibody-Mediated Prevention (AMP) study^9^ panel (most representative of circulating clade C HIV-1 strains) and HIV-1 strains of different co-receptor tropism. To assess BNT351’s developability on a large scale, which is needed for clinical testing, we analyzed potential sequence liabilities that could affect antibody integrity. Finally, we evaluated BNT351 activity in HIV-1-infected CD34^+^ humanized mice.

## Results

### BNT351 shows comparable target binding to 1-18 and no off-target binding

**Figure 1:**
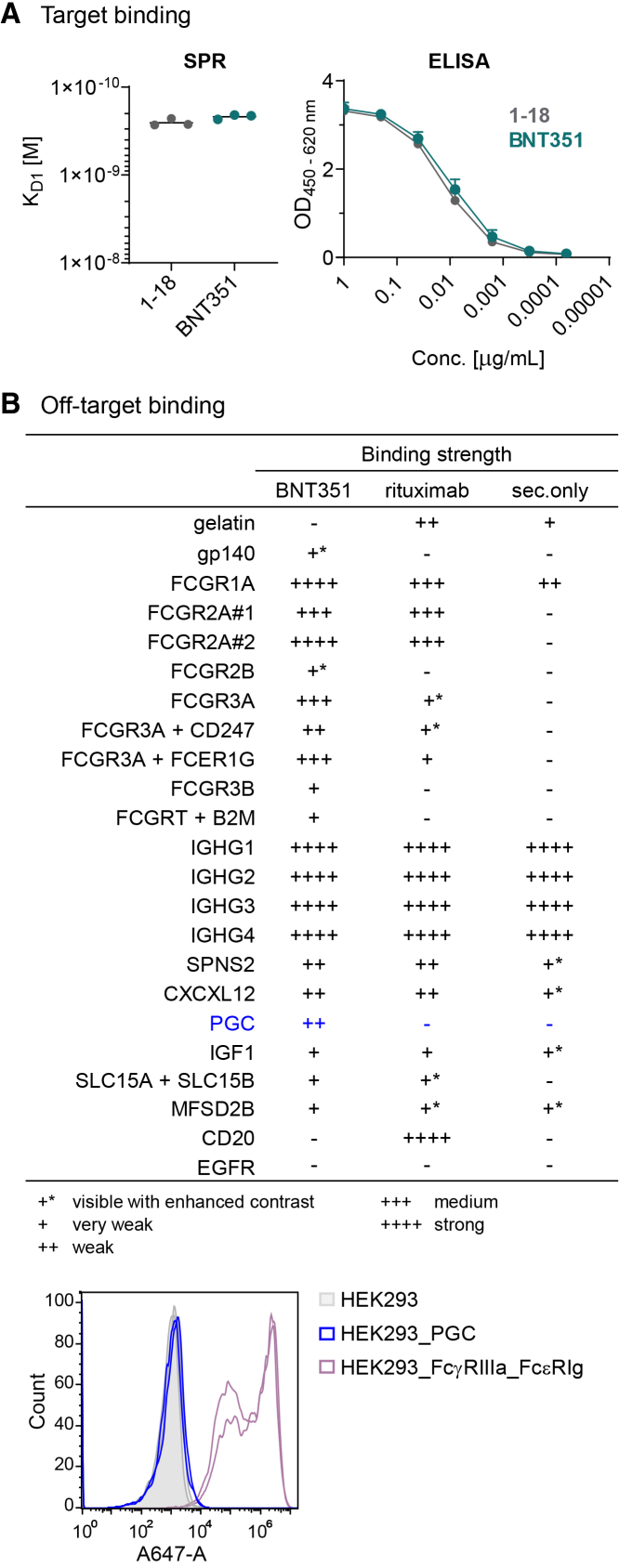
BNT351 shows comparable target affinity to 1-18 and no off-target binding (A) Left: the binding affinity of BNT351 and 1-18 to the stabilized HIV-1 envelope protein trimer (SOSIP_BG505.664_) by SPR (mean and individual values of triplicate measurements). Also see Figure S1A. Right: binding of BNT351 and 1-18 to SOSIP_BG505.664_ by ELISA (mean ± standard deviation of triplicate measurements). (B) BNT351’s off-target binding was assessed by Retrogenix Cell Microarray Technology platform. Interactions identified during the library screen with ∼6,500 human proteins are shown. Also, see Figure S2. Interaction with pepsinogen C (PGC) that appeared to be specific was re-tested by flow cytometry with HEK293 cells transfected with PGC. HEK293 cells transfected with FcγRIIIa and FcεRIg were used as a positive control. Duplicate measurements are shown for flow cytometry.

To confirm that LS mutations did not alter target binding, we compared the binding of BNT351 and its parental antibody, 1-18, to a stabilized HIV-1 Env trimer (SOSIP_BG505.664_)^10^ via surface plasmon resonance (SPR) and enzyme-linked immunosorbent assay (ELISA). 1-18 and BNT351 showed comparable, high affinity binding to SOSIP_BG505.664_ (*K*_D1_ of 0.26 nM for 1-18 and 0.22 nM for BNT351) (Figure 1A left; Figure S1A) and overlapping binding curves (Figure 1A right). In addition, we confirmed that 1-18 and BNT351 neutralized strain BG505.T332N with comparable potency (Tables S1 [IC_50_] and S2 [IC_80_]).

To exclude off-target binding of BNT351, we performed *in vitro* off-target profiling in the Retrogenix cell microarray. The library screen was done with HEK293 cells overexpressing ∼6,500 individual full-length human proteins, encompassing plasma membrane proteins, secreted proteins, and cell surface-tethered secreted proteins (∼6,100 monomers and 400 heterodimers). A rituximab biosimilar and a secondary antibody alone were used as controls to identify non-specific interactions. Slides spotted with CN54gp140 (Env of HIV-1 strain 97/CN/54) in gelatin served as positive control for BNT351 binding, HEK293 cells overexpressing CD20 served as positive control for rituximab biosimilar binding, and HEK293 cells overexpressing EGFR served as negative control for all tested antibodies. Although spotting a soluble protein in gelatin may lead to suboptimal protein accessibility, we observed a weak binding signal of BNT351 to CN54gp140, validating the target specificity.

BNT351 showed interaction with 19 human proteins out of ∼6,500 tested, most of which were human FcRs or targets of the secondary antibody used in the assay (Figure 1B top; Figure S2). The only BNT351-specific interaction (not seen for the rituximab biosimilar or secondary antibody) was with pepsinogen C (PGC) in the library screen; however, PGC binding was not seen in confirmatory flow cytometry analysis (Figure 1B bottom). Thus, we found no evidence of off-target binding for BNT351.

### BNT351 exhibits potent neutralization activity

**Figure 2:**
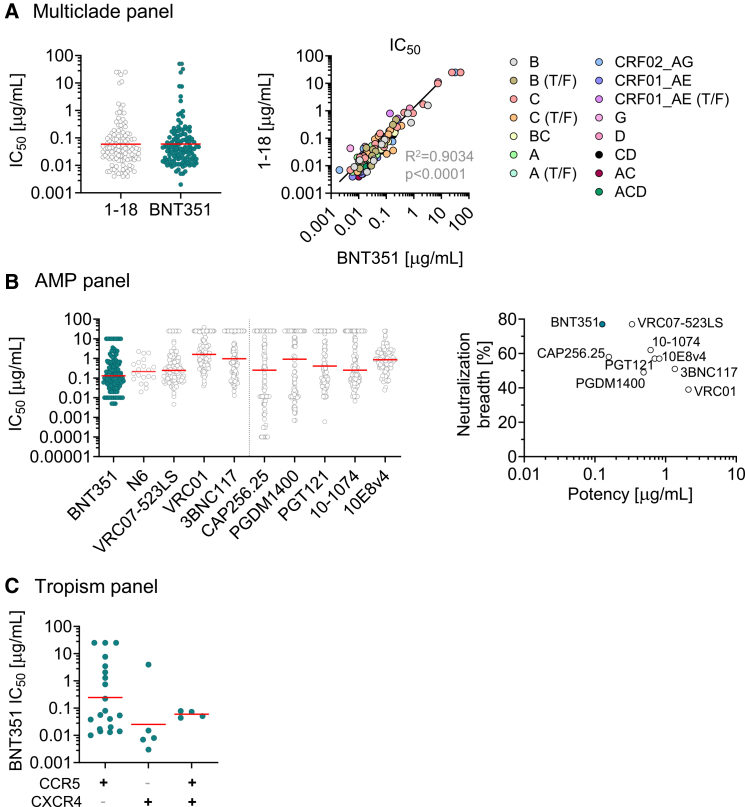
BNT351 shows highly potent neutralizing activity, comparable to the parental bNAb 1-18 Pseudovirus neutralization test (pVNT) with TZM-bl reporter cells was used to determine neutralization activity of BNT351 against (A) the multiclade panel (*n* = 119 strains), (B) an AMP panel of clade C strains (*n* = 106), and (C) the tropism panel covering HIV-1 strains of different co-receptor tropism (CCR5 [*n* = 20] or CXCR4 [*n* = 5] or dual [*n* = 4]). Following a 5-fold-7-step dilution of BNT351, IC_50_ values were determined. Red lines indicate geometric mean values. In (A) and (B) historical data for 1-18 and other bNAbs available in Los Alamos database are shown. Neutralization breadth was calculated using a threshold of IC_50_ < 1 μg/mL. Also see Tables S1–S4.

We evaluated the potential impact of the LS mutations on BNT351 neutralization activity. When tested against a multiclade HIV-1 pseudovirus panel of 119 strains,^11^ BNT351 potency (GeoMean IC_50_ = 0.053 μg/mL) and breadth (97%) highly correlated with those of 1-18 (R^2^ = 0.9034, *p* < 0.0001) (Figure 2A, Tables S1 [IC_50_] and S2 [IC_80_]).

To cover more contemporary HIV-1 strains, we tested BNT351 against an AMP panel, which is representative of circulating HIV-1 viruses identified in the AMP trials between 2016 and 2020.^9^^,^^12^ Specifically, we used 106 clade C strains identified in the placebo arm or the VRC01 treatment arm of the HVTN 703/HPTN 081 AMP trial. BNT351 exhibited the highest neutralization potency (IC_50_ = 0.13 μg/mL) and breadth (77%) against AMP circulating strains (Figure 2B and Table S3), compared with other clinically relevant HIV-1 bNAbs in our assay (Figure 2B).

Finally, to assess whether co-receptor tropism of HIV-1 strains affects neutralizing activity, we tested BNT351 against CCR5-tropic, CXCR4-tropic, and dual-tropic HIV-1 strains. Most of the tested HIV-1 strains were susceptible (GeoMean IC_50_ < 10 μg/mL) to BNT351, irrespective of their co-receptor tropism (Figure 2C). Only a few CCR5-tropic strains showed resistance to BNT351 (IC_50_ > 25 μg/mL): T27850, CAP45.2.00.G-3, and Du172.17. These BNT351-resistant strains remained susceptible to at least two of the three currently FDA-approved entry inhibitors (maraviroc, ibalizumab, and fostemsavir), suggesting no major risk of eliciting cross-resistance (Table S4).

### BNT351 shows low level of degradation under stress conditions

**Figure 3:**
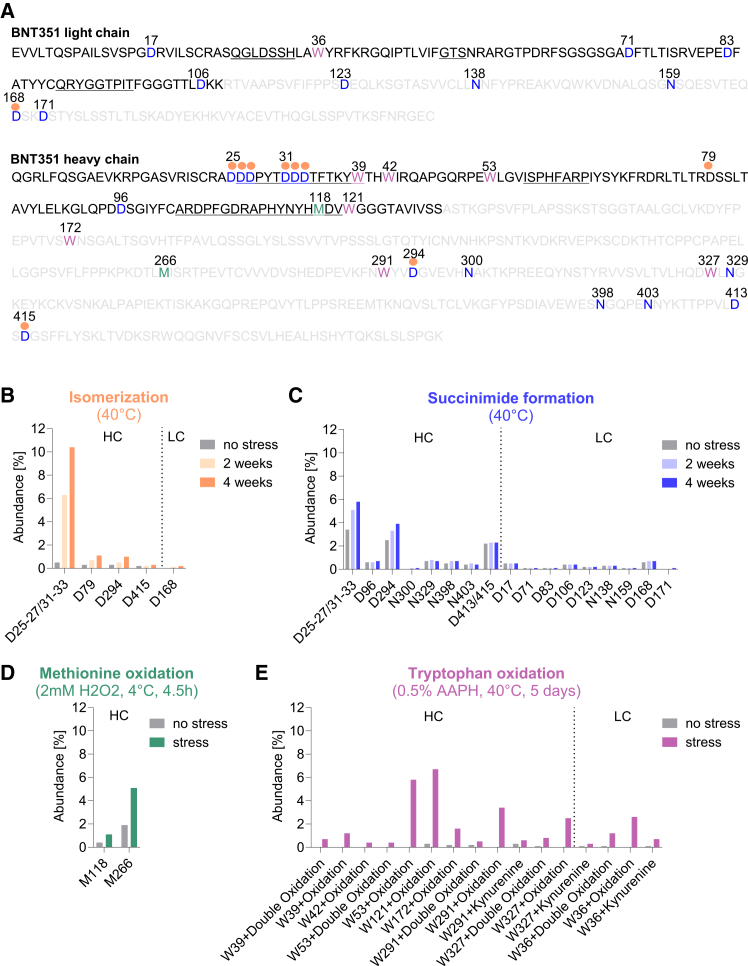
BNT351 shows high stability under stress conditions BNT351 was exposed to stress conditions (specified below), digested with trypsin or trypsin/LysC, and the obtained peptides were analyzed with liquid chromatography-tandem mass spectrometry (LC-MS/MS). (A) BNT351 amino acid sequence with variable regions in black letters, constant regions in gray letters, and complementarity-determining regions (CDRs) underlined. Residues analyzed for degradation are marked according to color scheme in (B)–(E). (B and C) Isomerization and succinimide formation after incubation at 40°C for 2 or 4 weeks. (D) Methionine oxidation under mild oxidative conditions (2 mM H_2_O_2_ at 2°C–8°C for 4.5 h). (E) Tryptophan oxidation under radical oxidative conditions (0.5% AAPH at 40°C for 5 days). Graphs in (B)–(E) show the abundance of degradation product at BNT351 residues indicated on *x* axes. For peptides containing multiple residues susceptible to degradation, the cumulative value is shown. BNT351 sample not exposed to stress conditions was used as reference (no stress). Also see Figure S3. HC, heavy chain; LC, light chain.

As part of developability testing before larger scale manufacturing, we conducted a series of stability tests. The aim was to analyze if certain amino acid sequence motifs present in BNT351, identified *in silico* as potential degradation hot spots, are susceptible to modification under accelerated stress conditions. Chemical modifications of special interest for antibodies are oxidation (of methionine and tryptophan), asparagine (Asn) deamidation (especially at the sequence motifs NG, NS, NT, and NN), and aspartate (Asp) isomerization (at sequence motifs like DG, DD, DT, and DS) as these modifications can lead to degradation. Of note, BNT351 features a six-amino acid insertion in the CDRH1 that contributes to its activity but is rich in aspartates, which can be prone to modification (Figure 3A). BNT351 stability testing included aspartic acid isomerization, aspartic acid/asparagine succinimide formation, methionine oxidation under mild oxidizing conditions, and tryptophan oxidation under radical oxidizing conditions. The approach consisted of exposing BNT351 to specific stress conditions, followed by digestion with trypsin, and peptides analysis with LC-MS/MS.

Isomerization levels of Asp/Asn residues were low after 4 weeks at 40°C (≤1.1% for peptides containing single Asp residues and 10.4% for the peptide containing multiple Asp residues) (Figure 3B). Similarly, succinimide formation after 4 weeks at 40°C was <6% (Figure 3C). BNT351 oxidation propensity was also low (<7%) after mild oxidative conditions (2 mM H_2_O_2_ at 2°C–8°C for 4.5 hours [h]) and radical oxidative conditions (0.5% 2,2′-azobis(2-amidinopropane) dihydrochloride [AAPH] at 40°C for 5 days) (Figures 3D and 3E). Taken together, BNT351 showed a low level of degradation under tested stress conditions, suggesting a favorable stability profile. Under storage conditions at 5°C, BNT351 preserved its binding potency to SOSIP_BG505.664_ at 12 months (Figure S3).

### BNT351 displays a favorable pharmacokinetic profile

**Figure 4:**
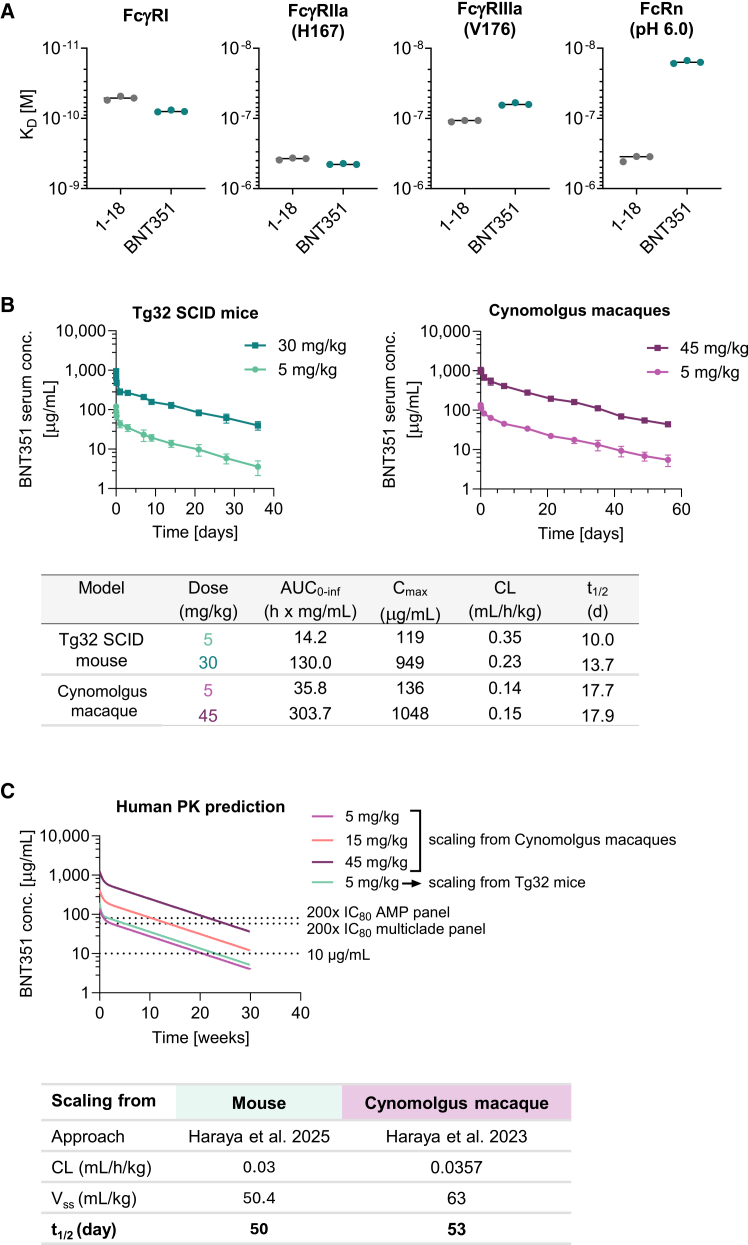
BNT351 shows higher affinity for FcRn than 1-18 and long half-life in Tg32 SCID mice and cynomolgus macaques (A) The binding affinity of BNT351 and 1-18 to human Fcγ receptors (FcγRs) and neonatal FcR (FcRn) by SPR. Mean and individual values of triplicate measurements are shown. H167 and V176 refer to the corresponding FcγR variant. Also see Figures S1B−S1F and S4. (B) BNT351 serum concentration in Tg32 SCID mice (*n* = 4/group) and in cynomolgus macaques (*n* = 3/group) injected intravenously with indicated doses of BNT351 on day 0. Mean values ±SD are shown. The table shows calculated pharmacokinetic (PK) parameters. Also see Figures S5 and S6 and Table S5. (C) Human PK projection using allometric scaling of PK parameters from Tg32.SCID mice (allometric exponents from Haraya et al.^13^) and cynomolgus macaques (allometric exponents from Haraya et al.^14^).

LS mutations increase the antibody’s binding affinity to human FcRn, without affecting binding to FcγRs. To confirm the effect of LS mutations, we evaluated interactions of BNT351 and 1-18 with human FcγRs (FcγRI/CD64, FcγRIIA/CD32a, and FcγRIIIA/CD16a) and FcRn via SPR analysis. While BNT351 and 1-18 displayed comparable affinity (*K*_D_) for hFcγRs, BNT351 showed higher binding and a slower dissociation rate to hFcRn compared to 1-18, resulting in a more than 20-fold higher hFcRn affinity at pH 6.0, at which endosomal recycling occurs (Figures 4A and S1B–S1E and Table 1). As expected, both 1-18 and BNT351 showed low affinity for hFcRn at pH 7.4 at which IgG antibodies are released from hFcRn to the circulation (Figures S1F and S4 and Table 1). In summary, LS mutations substantially increased BNT351’s affinity for hFcRn at pH 6.0 without altering binding to hFcγ receptors.

**Table 1:** Equilibrium dissociation constant (*K*_D_) of 1-18 and BNT351 with human Fc receptors (FcR)

	1-18 *K*_D_ [M] (mean ± SD)	BNT351 *K*_D_ [M] (mean ± SD)
FcγRI	(5.16 ± 0.33) × 10^−11^	(7.90 ± 0.26) × 10^−11^
FcγRII_H167_	(3.77 ± 0.14) × 10^−7^	(4.48 ± 0.08) × 10^−7^
FcγRIIIa_V176_	(10.9 ± 0.29) × 10^−8^	(6.26 ± 0.26) × 10^−8^
FcRn (pH 6)	(37.2 ± 3.7) × 10^−8^	(1.58 ± 0.08) × 10^−8^
FcRn (pH 7.4)	binding too low to calculate	(2.32 ± 0.02) × 10^−6^

To assess BNT351 pharmacokinetics and to inform the dose selection for clinical studies, we evaluated the PK profile following intravenous (IV) single-dose bolus administration of BNT351 in two animal models: SCID FcRn-/- hFcRn (32) transgenic (Tg) mice (Tg32 mice) and cynomolgus macaques. Tg32 mice express human FcRn instead of the murine FcRn, allowing for more accurate half-life prediction for human IgG antibodies than wild-type mice,^15^ while the SCID background lowers the risk for immunogenicity against the test item.^16^ In both PK studies, BNT351 serum concentrations were measured with a sandwich immunoassay (MSD) using an anti-idiotypic antibody specific to BNT351.

In Tg32 mice, two BNT351 dose levels were tested (5 mg/kg and 30 mg/kg) resulting in a half-life of 10–14 days (Figure 4B). The increase in systemic exposure (area under the curve [AUC] and maximum observed concentration [C_max_]) from 5 to 30 mg/kg was not dose proportional. Similarly, two dose levels were tested in cynomolgus macaques (5 and 45 mg/kg). One animal in the 45 mg/kg dose group showed drastically decreased serum BNT351 concentration after 168 h, likely due to the development of anti-drug antibodies (ADAs), and was excluded from the analysis (Figure S5). In cynomolgus macaques, BNT351 showed an approximately 2-fold lower clearance than a typical non-LS-engineered mAb,^17^ and a half-life of 18 days (Figure 4B). AUC_0-inf_ and C_max_ increased dose-proportionally from 5 to 45 mg/kg. In both animal models, BNT351 exhibited a volume of distribution in the range of the anatomical plasma volume, indicating a plasma-restricted distribution (Table S5).

To predict human half-life, we developed a population PK model for both species based on the measured serum concentrations, to derive respective typical two-compartment PK parameters. We then performed allometric scaling of these parameters to humans using published exponents for LS-engineered IgG antibodies for Tg32 mice^13^ and cynomolgus macaques.^14^ The analysis predicted a human terminal half-life of BNT351 in the range of 50–53 days (Figure 4C), which aligns with published data on other LS-engineered broadly neutralizing antibodies.^18^

Next, we performed PK projections to estimate for how long a given BNT351 dose would maintain plasma antibody concentrations above expected protective thresholds in humans (Figure 4C). For the frequently used threshold of 10 μg/mL, below which rebound was observed for other bNAbs,^2^ the PK simulations projected a single IV dose of 5 mg/kg BNT351 to sustain coverage for ∼5 months. Subsequently, we applied a more stringent threshold of 200-fold over geomean IC_80_, proposed for protection efficacy,^4^ basing our calculations on IC_80_ values against the multiclade panel (0.29 μg/mL x 200 = 58 μg/mL) and the AMP panel (0.4 μg/mL x 200 = 80 μg/mL) (Figure 2). Under this scenario, a single IV dose of 45 mg/kg BNT351 was predicted to sustain coverage for 5–6 months.

It has been reported that target-mediated drug disposition (e.g., viral load) can have an impact on the PK of bNAbs in humans.^19^ We measured BNT351 serum concentrations in HIV-1_YU2_-infected CD34^+^ humanized NSG mice (humice) to test this hypothesis. BNT351 serum levels were comparable between HIV-1_YU2_-infected and non-infected mice (Figure S6), suggesting no major impact of viremia on BNT351 PK in this model.

### BNT351 suppresses viremia *in vivo* without escape

**Figure 5:**
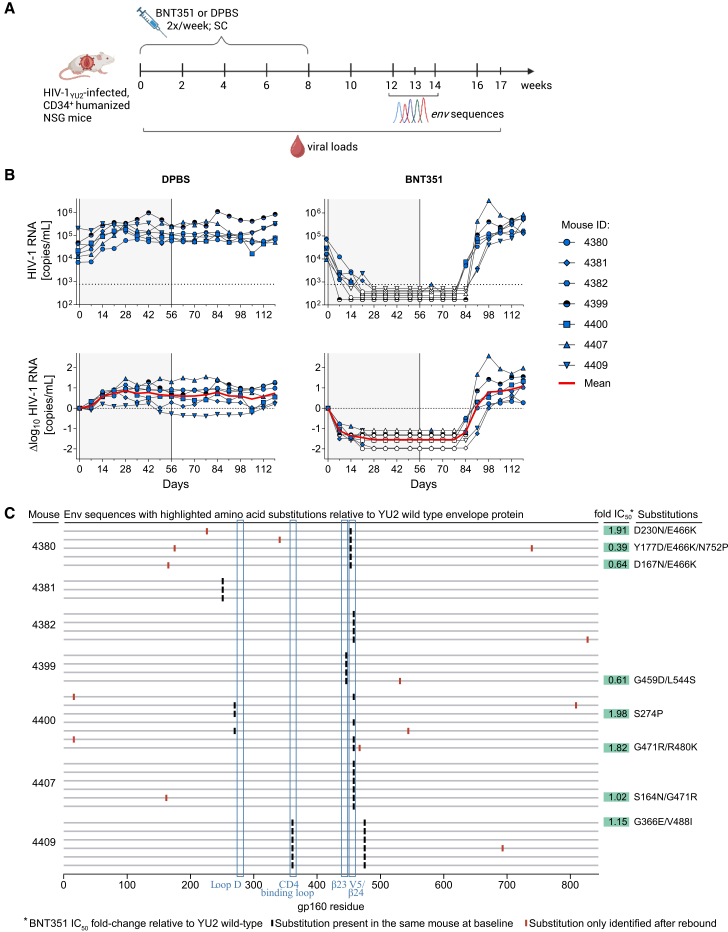
BNT351 suppresses viremia in HIV-1-infected humanized CD34^+^ NSG mice without selecting for resistant viral variants (A) Experiment scheme: HIV-1_YU2_-infected CD34^+^ humanized NSG mice (*n* = 7/group) received subcutaneous (SC) injections of BNT351 (1 mg loading dose, followed by 0.5 mg every 3–4 days for 8 weeks) or DPBS (control) on the same days. Plasma viral loads were monitored once weekly for 17 weeks, and plasma SGS was performed at baseline and on day 84, 91, or 98. (B) RT-qPCR was used to measure plasma viral loads. HIV-1 RNA plasma copies (top) and log10 viral load changes compared with baseline (bottom) are shown. Gray shading indicates the BNT351 treatment period. Data points in white indicate viral loads below lower limit of quantification (LLOQ = 774 copies/mL). For visualization in the absolute copy number plots, viral loads <LLOQ were assigned an arbitrary value so that lines and icons of individual mice remained distinguishable. For calculation and visualization of log10 changes compared to baseline, a value of 773 was assigned to all viral load measurements <LLOQ. Red lines show average log_10_ viral load change compared with baseline. (C) Plasma *env* sequences obtained by SGS in individual mice after viral rebound (day 84, 91, or 98). Blue boxes highlight key regions of potential escape from CD4 binding site antibodies. Bars indicate amino acid substitutions relative to HIV-1_YU2_ wild type already identified at baseline in the same mouse (black) or only identified after rebound (red). A selection of SGS-derived *env* sequences containing indicated mutations were produced as pseudoviruses and their sensitivity to BNT351 tested in a pseudovirus neutralization test with TZM-bl reporter cells. IC_50_-fold changes to wild-type sequence <2.5 indicate BNT351 sensitivity. Note: the x-axis numbering is according to HIV-1_YU2_ strain, while the substitutions are numbered according to the (conventional) HIV-1_HXB2_ numbering scheme.

We used HIV-1_YU2_-infected humice to evaluate BNT351 antiviral activity. Neutralization activity of BNT351 against YU2 strain was confirmed prior to the *in vivo* experiment (Tables S1 [IC_50_] and S2 [IC_80_]). Mice with confirmed viremia received a 1 mg loading dose of BNT351 subcutaneously (SC), followed by twice weekly SC injections of 0.5 mg of BNT351 for 8 weeks (Figure 5A). The control group received Dulbecco’s phosphate buffered saline (DPBS) on the same days.

While viremia was not suppressed in control humice, monotherapy with BNT351 resulted in sustained viral suppression during treatment and up to 5 weeks after treatment cessation (HIV-1 RNA average drop of 1.5 log_10_ copies/mL) (Figure 5B). In all BNT351-treated animals, viremia dropped below the lower level of quantification (774 copies/mL) by day 28. Overall, antiviral activity of BNT351 was comparable to the parental bNAb 1-18.^3^

Four to six weeks after BNT351 treatment cessation (days 84, 91, or 98), plasma single genome sequencing (SGS) of rebound viruses was performed to determine whether declining BNT351 levels and decreased immune pressure contributed to the emergence of variants carrying escape mutations (Figure 5C). SGS analyses did not show a recurrent pattern of mutations in expected contact residues on the Env of YU2 strain (Figure 5C left, mutations identified after rebound shown in red). To confirm BNT351 sensitivity of viruses that rebounded post-treatment, we produced pseudoviruses using SGS-derived *env* sequences from distinct mice with different gp160 amino acid substitutions (Figure 5C, right). All rebound viruses remained sensitive to BNT351, showing similar susceptibility as wild-type YU2 pseudovirus (Figure 5C, right).

In conclusion, BNT351 fully suppressed viremia in HIV-1-infected humanized CD34^+^ NSG mice without selecting for resistant viral variants.

## Discussion

Here, we report the preclinical assessment of the next-generation HIV-1 bNAb, BNT351, in preparation of clinical development. BNT351 has the original Fab fragment of the parental bNAb 1-18^3^ and has been engineered for extended half-life by incorporating the LS mutations into the Fc region.

SPR analysis confirmed increased affinity of BNT351 for hFcRn compared with 1-18, in line with findings for other LS-modified antibodies.^20^ The improvement was reflected in subsequent *in vivo* PK studies in both Tg32 mice and cynomolgus macaques, where BNT351 exhibited favorable PK profiles with a projected half-life in humans of approximately 50 days. While the half-life needs to be confirmed in future clinical trials, the PK projection compares favorably to reported half-lives for other CD4bs-targeting HIV-1 bNAbs such as VRC07-523LS (estimated mean half-life of 42 days across all doses and routes)^21^ and N6LS (serum half-life of 48.6 days).^22^ Prolonged half-life of BNT351 could enable less frequent dosing^18^—once every 2 to 6 months based on our projections—thereby reducing the treatment burden. ADA formation likely occurred in one out of six cynomolgus macaques during the PK evaluation of BNT351. Such immune responses are frequently reported in studies administering human antibodies to animals and are not considered predictive of immunogenicity in humans.^23^

Some bNAbs show a reduced half-life in individuals with HIV-1-viremia, possibly due to accelerated clearance of antigen-antibody complexes,^19^^,^^24^ while others show unchanged PK profiles.^25^ In a humice model, we observed no differences between BNT351 PK profiles in the presence or absence of HIV-1. Since PK analyses in the humice may not be fully translatable to humans, clinical data are needed to confirm if BNT351 exhibits comparable PK profiles in people living with HIV-1 and without HIV-1.

Notably, the LS mutations extended BNT351’s half-life without altering its functionality. We confirmed that BNT351 maintained the target affinity, FcγR affinity, and neutralization potency and breadth of parental bNAb 1-18. In a confirmatory study using a humice HIV-1 infection model, BNT351 fully suppressed viremia. Importantly, *de novo* mutations in rebound viruses were mostly identified outside BNT351’s binding sites, did not show recurring patterns within and across mice, and did not affect BNT351’s neutralization activity. This suggests random mutations through the error-prone reverse transcriptase, rather than immunologically favorable selection. As infection models with a monoclonal virus, such as used in this study, cannot fully recapitulate the diversity of HIV-1 seen in humans, clinical trials will be important to further assess the risk of mutational escape to BNT351. Observed *in vivo* activity of BNT351 was comparable to previously reported data for 1-18.^3^ This was expected since humice do not express human FcRn in endothelial cells, necessary for the active recycling of the LS-modified antibodies. Therefore, the therapeutic advantage of BNT351 due to a longer half-life cannot be assessed in this model.

The expanded neutralization data from the tropism panel showed that BNT351 can neutralize HIV-1 strains irrespective of their co-receptor tropism. Using the same tropism panel, we showed no major risk of cross-resistance between BNT351 and FDA-approved entry inhibitors. While encouraging, this finding requires further validation by reciprocal testing of more BNT351- and entry inhibitors-resistant strains. When assessed against clade C strains from the AMP panel, which is representative of contemporary viral strains with increased resistance to CD4bs-targeting bNAbs,^12^ BNT351 outperformed clinically tested CD4bs-targeting bNAbs VRC01 and 3BNC117 and showed comparable activity to VRC07-523LS. This is in line with results from recent neutralization studies against primary African isolates^26^ and Indian clade C isolates,^27^ in which 1-18 and VRC07-523LS showed higher activity than VRC01.

In addition to antibody activity, other aspects such as developability and toxicity play an important role in antibody development. Antibody developability is critical for efficient large-scale manufacturing and should be ensured during preclinical testing. Our stability tests under stress conditions did not identify developability risks for BNT351. Importantly, the unusual six-amino-acid insertion in BNT351’s CDRH1^3^ did not show increased degradation levels. Finally, off-target profiling in a cell microarray using ∼6,500 human proteins did not reveal any off-target binding for BNT351. This lack of BNT351 cross-reactivity to human proteins builds upon previously published data showing no binding of 1-18 to human Hep-2 cells.^3^ Toxicities related to cross-reactivity are, therefore, not anticipated.

In summary, bNAb BNT351, featuring LS mutations in the Fc region, showed extended half-life in animal models, while maintaining the exceptional antiviral *in vitro* and *in vivo* activity of its parental bNAb 1-18. Taken together with the favorable developability assessment, no off-target binding, low viral escape risk, and no cross-resistance with entry inhibitors, we selected BNT351 as a suitable candidate for clinical development. The ongoing phase 1 clinical trial will assess its safety and antiviral activity in human populations (NCT07392372).

### Limitations of the study

To show BNT351’s antiviral activity *in vivo*, we used recombinantly produced monoclonal HIV-1_YU2_ strain in a humanized mouse model. While this model allows HIV-1 diversification *in vivo*,^28^ it does not fully capture the diversity of viruses present in people living with HIV. Clinical evaluation is, therefore, needed to fully characterize BNT351’s antiviral activity. Similarly, although we used the best available preclinical models to evaluate PK, it will be important to confirm the half-life in humans, especially in a therapeutic setting where active viral clearance by opsonization could impact antibody titer. Contribution of BNT351’s Fc domain to the mode-of action also requires further evaluation, as we only evaluated its binding to human FcRs but not the downstream cellular effects such as ADCP and ADCC. Lastly, we only assessed BNT351 as monotherapy, but a combination with other bNAbs or antivirals may be needed in a human therapeutic setting.

## Resource availability

### Lead contact

Requests for further information and resources should be directed to and will be fulfilled by the lead contact, Sven Kratochvil (sven.kratochvil@biontech.de).

### Materials availability

BNT351 and anti-idiotypic antibody against 1-18/BNT351 are available to academic, non-commercial researchers with a completed materials transfer agreement upon reasonable request.

### Data and code availability

The 1-18 sequence has been reported before.^3^ The BNT351 protein sequence is provided in Figure 3. SGS-derived HIV-1 *env* sequences obtained from HIV-1_YU2_-infected humanized mice have been deposited at GenBank: PX492161-PX492221. All data associated with this study can be found in the main text or the supplemental information. Source data are available upon reasonable request. This paper does not report original code. Any additional information required to reanalyze the data reported in this paper is available from the lead contact upon reasonable request.

## Acknowledgments

We thank A. Plaschke, A. Trapp, S. Krapp, and L. Fischer for assay development; R. Shetge, R. Wagner-Uhler from 10.13039/100032339BioNTech CMC unit for overseeing antibody production; K. Hampel, M. Zenn, E. Franz, and B. Zimmermann from Biaffin GmbH & Co KG for SPR analyses; K. Siddals, G. Sixsmith, and H. Thomas from Charles River for off-target profiling; S. Maier and A. Zeck from Natural and Medical Sciences Institute (NMI, Germany) for stability studies; staff of Evotec and Charles River Laboratories for PK studies; Anna Schmitt for humanized mouse experiment regulatory support and the staff of the Animal Care Facility Weyertal at the 10.13039/501100008001University of Cologne; J. Nieβl for reviewing the manuscript. Figures 5A and S4A were created with BioRender.com. This study was funded by 10.13039/100032339BioNTech SE.

## Author contributions

S.K., V.L.D., and U.Ş. conceived the study; S.K., M.K., H.G., C.-H.T., C.J., C.L., S.P., F.T., P.S., N.M., P.M., M.S., F.K., and V.L.D. designed the experiments; H.G., S.S., S.P., R.S., J.K. J.N., A.M., and S.B. performed experiments; S.K., M.K., H.G., S.S., C.-H.T., N.V., F.T., J.N., P.S., S.B., M.S., and V.L.D. analyzed the data; S.K., M.K., H.G., S.S., N.V., C.J., C.L., S.P., F.T., P.S., N.M., P.M., F.K., and V.L.D. interpreted the data; S.K., N.V., and V.L.D. were responsible for manuscript preparation and revision. All authors reviewed and approved the final manuscript.

## Declaration of interests

S.K., M.K., C.-H.T., N.V., C.J., C.L., S.P., F.T., U.Ş., J.N., A.M., and V.L.D. are employees at BioNTech group and may hold stock options. U.Ş. is a management board member and stock owner of BioNTech SE (Mainz, Germany). BioNTech SE has a strategic relationship with the Gates Foundation to develop HIV vaccines and immunotherapies, but no specific support was received for the work reported here. H.G. holds Togontech GmbH stock options and has received consulting fees from GSK. H.G., P.S., and F.K. are inventors on the International Patent Application related to 1-18. S.K., M.K., C.-H.T., and V.L.D. are inventors on the International Patent Application related to BNT351. S.K., F.T., J.N., and V.L.D. are inventors on patents related to RNA-encoded anti-HIV antibodies. U.Ş. and V.L.D. are inventors on the patent related to RNA vaccine against HIV.

## STAR★Methods

### Key resources table

**Table:** 

REAGENT or RESOURCE	SOURCE	IDENTIFIER
**Antibodies**		

1–18	This paper; Schommers et al.^3^	N/A
BNT351	This paper	N/A
Rituximab biosimilar (Retrogenix)	Charles River	N/A
Biotinylated anti-idiotypic (anti-1-18 F(ab')_2_) antibody	Bio Rad AbD Serotec, GmbH	N/A (custom-made)
Ibalizumab	Dr. David Ho, Aaron Diamond AIDS Research Center	N/A

**Bacterial and virus strains**		

119 multiclade panel	M.S. Seaman, BIDMC	N/A
AMP panel (clade C)	N.N. Mkhize and P.L. Moore	N/A
Tropism panel	M.S. Seaman, BIDMC	N/A
Replication-competent recombinant HIV-1 (YU2 Env in NL4-3 backbone)	Zhang et al.^29^	N/A

**Chemicals, peptides, and recombinant proteins**		

MabSelect SuRe Protein A resin	Cytiva	Cat#17-5438-02
EZ-Link^TM^ Sulfo-NHS-LC-Biotin	Thermo Fisher	Cat#21335
human FcγRI/CD64	Acro Biosystems	Cat#FCA-H82E8
human FcγRIIIA/CD16a (V176)	Acro Biosystems	Cat#CDA-H82E6
human FcγRIIA/CD32a (H167)	Acro Biosystems	Cat#CDA-H82E6
human FcRn/FCGRT&B2M	Acro Biosystems	Cat#FCM-H82W7
SOSIP_BG505.664_ Env trimer	ATUM	Custom-made
Casein blocking buffer	Sigma-Aldrich	Cat#B6429
3,3′,5,5′-tetramethylbenzidine (TMB) substrate	Biotrend Chemikalien GmbH	Cat#4380A
CN54gp140	Polymun	#ENV002
FuGENE® 6 reagent	Promega	Cat#E2691
Phusion® Hot Start Flex DNA Polymerase	New England Biolabs	Cat#M0535L
Maraviroc	NIH AIDS Reagent program	Cat# HRP-11580
Fostemsavir	Sigma-Aldrich	Cat#TA9H11E419D1
DEAE-dextran	Sigma-Aldrich	Cat#D9885
Bright-Glo™ luciferase assay system	Promega	Cat#E2650
ATP	Sigma-Aldrich	Cat#A26209; CAS: 34369-07-8
Coenzyme A	Sigma-Aldrich	Cat#C3144; CAS: 55672-92-9
IGEPAL	Sigma-Aldrich	Cat#I8896; CAS: 9002-93-1
D-Luciferin	GoldBio	Cat#LUCNA; CAS: 103404-75-7
Trypsin	Promega	Cat#V528A
Trypsin/LysC	Promega	Cat#V5073
L-methionine	Sigma-Aldrich	Cat#64319; CAS: 63-68-3
AAPH (2,2′-Azo-bis(2-methylpropionamidine) -dihydrochloride)	Sigma-Aldrich	Cat#440914; CAS: 2997-92-4
Guanidine hydrochloride	Carl Roth	Cat#0037.2; CAS: 50-01-1
DTT (1,4-Dithiothreitol)	Carl Roth	Cat#6908.4; CAS: 3483-12-3
Trifluoroacetic acid (ULC-MS-Ortigrade LGC)	Promochem	Cat#SO-9668-B000; CAS: 76-05-1
L-Histidine hydrochloride monohydrate	Carl Roth	Cat#1697.2; CAS: 5934-29-2
TCEP (Tris-(2-carboxyethyl)-phosphine hydrochloride)	Carl Roth	Cat#HN95.2; CAS: 51805-45-9
Formic acid	Promochem	Cat#SO-9679-B005; CAS: 64-18-6
Acetonitrile	Carl Roth	Cat#AE70.2; CAS: 75-05-8
Superscript™ IV reverse transcriptase	Thermo Fisher	Cat#18090050
RNase H	Thermo Fisher	Cat#18021071
Platinum™ Taq Green Hot Start DNA Polymerase	Thermo Fisher	Cat#11966034

**Critical commercial assays**		

ExpiCHO™ transfection system	Thermo Fisher	Cat#A29133
Gyrolab Bioaffy 20 HC CD	Gyros Protein Technologies	Cat#P0004424
Gyrolab 2G Generic PK Type F Kit Reagents	Gyros Protein Technologies	Cat#P0020958
MinElute® Virus Mini Spin Kit	Qiagen	Cat#57704
TaqMan™ RNA-to-CT™ 1-Step-Kit	Thermo Fisher	Cat#4392938
Cobas® 6800 HIV-1 kit	Roche	Cat#09040803190

**Deposited data**		

Mouse plasma SGS-derived HIV-1 *env* sequences	This paper	GenBank: PX492161-PX492221

**Experimental models: Cell lines**		

ExpiCHO™ cells	Gibco	Cat#A29127
HEK293T cells	ATCC	Cat#CRL-11268
TZM-bl cells	NIH AIDS Reagent Program	Cat#8129

**Experimental models: Organisms/strains**		

Tg32 mice (B6.Cg-*FcgRt*^*tm1Dcr*^*Prkdc*^*scid*^ Tg(FCGRT)32Dcr/DcrJ	The Jackson Laboratory	RRID:IMSR_JAX:018441
Cynomolgus macaques (*Macaca fascicularis*)	Noveprim	N/A
Humanized CD34^+^ NSG female mice (NOD.Cg-Prkdc^scid^ Il2rg^tm1Wjl^/SzJ	The Jackson Laboratory	RRID:IMSR_JAX:005557

**Oligonucleotides**		

Primer env1Atopo 5’-CACCGGCTTAGGCAT CTCCTATGGCAGGAAGAA	IDT	N/A
Primer envN 5′-CTGCCAATCAGGGAAGTAGCCTTGTGT	IDT	N/A
CMVenv 5′-AGTAATCAATTACGGGGTCATTAGTTCAT	IDT	N/A
Primer Rev19 ACTTTTTGACCACTTGCCACCCAT	IDT	N/A
Primer 5′-TAATGGCAGCAATTTCACCA	IDT	N/A
Primer 5′-GAATGCCAAATTCCTGCTTGA	IDT	N/A
Primer YB383 5′TTTTTTTTTTTTTTTTTTTTTTTTRAAGCAC	IDT	N/A
Primer YB50 5′-GGCTTAGGCATCTCCTATGGCAGGAAGA	IDT	N/A
Primer YB49 5′-TAGAAAGAGCAGAAGACAGTGGCAATGA	IDT	N/A
Primer YB52 5′ GGTGTGTAGTTCTGCCAAT CAGGGAAGWAGCCTTGTG	IDT	N/A

**Recombinant DNA**		

pcDNA™3.4TOPO™ expression vectors encoding corresponding heavy and light chains of 1–18 or BNT351	This paper	N/A
pIRES-insert-IRES-ZsGreen1 expression vectors encoding human proteins	Retrogenix	N/A
YU2 Env expression plasmid	M.S. Seaman, BIDMC	N/A
*Env*-deficient HIV-1 backbone vector (pSG3Δenv)	NIH AIDS Reagent Program	Cat#11051

**Software and algorithms**		

Biacore T200 Evaluation Software (version 3.2)	Cytiva	https://www.cytivalifesciences.com/en/de/support/software/biacore-downloads/biacore-t200-software
ImageQuant (version 8.2)	GE healthcare (now part of Cytiva)	https://www.cytivalifesciences.com/en/us/products/items/imagequant-tl-analysis-software-p-28619?psmenu=2
Phoenix® NLME (version 8.3.5.340)	Certara	https://www.certara.com/software/phoenix-nlme/
Gyrolab Evaluator	Gyros Protein Technologies	https://www.gyrosproteintechnologies.com/immunoassays/gyrolab-systems/software

**Other**		

HiLoad Superdex™ 200 pg 26/60 column	Sigma-Aldrich	Cat#GE28-9893-36
ACQUITY Peptide BEH C18 Column	Waters	Cat#186005594

### Experimental model and study participant details

#### Cell lines

ExpiCHO cells (Gibco) were maintained in a shaking incubator at 36.8°C, 8% CO_2_, a relative humidity of 85% in ExpiCHO Expression Medium (Gibco). HEK293T cells (ATCC) were maintained at 37°C and 5% CO_2_ in Dulbecco’s Modified Eagle Medium (DMEM, Thermo Fisher) supplemented with 10% fetal bovine serum (FBS, Sigma-Aldrich), 1 mM sodium pyruvate, 2 mM L-glutamine, and 1× antibiotic-antimycotic (all from Thermo Fisher). TZM-bl cells (NIH AIDS Reagent Program) were maintained at 37°C in 5% CO_2_ in DMEM (Thermo Fisher) supplemented with 10% FBS (Sigma-Aldrich), 1 mM sodium pyruvate (Thermo Fisher), 2 mM L-glutamine (Sigma-Aldrich), 50 μg/mL gentamicin (Sigma-Aldrich), and 25 mM 4-(2-hydroxyethyl)-1-piperazineethane-sulfonic acid (HEPES, Thermo Fisher or Sigma-Aldrich). The sex of ExpiCHO, HEK293T and TZM-bl cell lines is female. Cell lines were not specifically authenticated. All cell lines were routinely tested for mycoplasma contamination, except for HEK293T and TZM-bl for neutralization assay against strains YU2 and BG505.T332N.

#### *In vivo* studies design

Statistical methods were not used to predetermine group sizes. Group sizes for the treatment experiment (*n* = 7/group; total of 14 mice) were selected based on historical data for 1–18 and the expected outcome (suppression in all BNT351-treated mice versus maintained viremia in control mice). Mice were assigned to groups based on their baseline viral loads to ensure comparable baseline viremia. For the PK studies in Tg32 mice and cynomolgus macaques, the minimum number of animals deemed adequate to meet the study objectives was selected based on experience from comparable studies (Tg32: *n* = 4/group, total of 8 mice; cynomolgus macaques: *n* = 3/group, total of 6 animals). Animals were not randomized. Group sizes for the PK study in humanized mice were based on availability of infected and uninfected humanized mice (*n* = 8/group; total of 16 mice).

Investigators were not blinded during experiments or analysis. Each *in vivo* experiment was performed once. Group sizes and replicate numbers are specified in corresponding Figure legends. In all studies, groups were age- and sex-matched. No other confounders were controlled.

#### Animal models, husbandry and ethics

Male Tg32 mice (B6.Cg-*FcgRt*^*tm1Dcr*^ *Prkdc*^*scid*^ Tg(FCGRT)32Dcr/DcrJ; JAX Strain 018441), with an approximate age of 10 weeks at study start, were housed at Evotec France SAS animal facility in plastic cages under controlled environmental conditions (20°C–24°C, 40–60% relative humidity, 12–15 air changes (AC)/h). Safe diet AO4C (Safe Diets) and tap water were provided *ad libitum*. The study was carried out under an approved animal-use protocol (APAFIS#24998–2020040623463195 v5) that had been reviewed by the Evotec France Ethical Committee and authorized by the French Ministry of Education, Advanced Studies and Research. The study was reviewed by the SBEA (Evotec internal Animal Welfare Body). Evotec animal facility is accredited by the French Ministry of Agriculture and by the Association for Assessment and Accreditation of Laboratory Animal Care International (AAALAC).

Male cynomolgus macaques (*Macaca fascicularis*), with an approximate age of 22–36 months at study start, were group housed in dedicated cages under controlled conditions (20°C–24°C, 30–70% relative humidity, 8–155 AC/h). For psychological/environmental enrichment, animals were provided with at least four items such as toys, except when interrupted by study procedures/activities. Primate-Leaf eater vegetarian diet (ssniff) was provided once daily in amounts appropriate for the size and age of the animals. Tap water was available *ad libitum*. The Charles River Laboratories Evreux Ethics Committee (CEC) reviewed the Study Plan to assess compliance with the corresponding authorized project as defined in Directive 2010/63/EU and in French decree No. 2013-118. The study was conducted in compliance with Council Directive No. 2010/63/EU and French decree No. 2013-118 on the protection of animals used for scientific purposes. The test facility is AAALAC accredited.

Humanized CD34^+^ NSG female mice (NOD.Cg-Prkdc^scid^ Il2rg^tm1Wjl^/SzJ, JAX strain #005557) engrafted with human cord blood-derived CD34^+^ hematopoietic stem cells) were purchased from The Jackson Laboratory and maintained at the Decentralized Animal Husbandry Network (*Dezentrales Tierhaltungsnetzwerk*) of the University of Cologne under specific pathogen-free (SPF) conditions until the start of experiment (HIV-1 challenge), when they were moved to an S3∗∗ facility. Mice were 22 weeks old at study start. Mice were kept in individually ventilated cages (maximum five animals per cage) and under controlled environmental conditions (20°C–22°C, 50–60% relative humidity, 75 AC/h). Cages contained dust-free bedding made of debarked chopped aspen wood and additional nesting material. Gamma-irradiated ssniff phytoestrogen-reduced food (ssniff Spezialdiäten GmbH) and autoclaved tap water were provided *ad libitum* and changed at least once weekly. Humanized mouse experiments were authorized by the State Agency for Nature, Environmental Protection, and Consumer Protection North Rhine-Westphalia (LANUV; approval number 81-02.04.2021.A050) and conducted at the University of Cologne according to the Federation of European Laboratory Animal Science Associations (FELASA) recommendations and in compliance with the German animal welfare act and Directive 2010/63/EU.

In all studies, only animals with an unobjectionable health status were selected for testing procedures after acclimatization period, and humane endpoints were predefined.

### Method details

#### Recombinant antibody production

1-18 was produced by transient transfection in ExpiCHO cells. BNT351 was produced from a stably transfected cell clone in large bioreactors at WuXi Biologics GMP facility (China) or by transient transfection in ExpiCHO cells. For transient transfection, ExpiCHO suspension cells were co-transfected with pcDNA3.4TOPO expression vectors encoding corresponding heavy and light chains using the ExpiCHO transfection system (Thermo Fisher). Cells were incubated at 36.8°C, 8% CO_2_, a relative humidity of 85%, and 75 rpm shaking speed. Twenty-four hours after transfection, enhancer and feed medium were added to the cells according to the manufacturer’s protocol. Eight days after the transfection, cell culture supernatant was harvested in a 2-step centrifugation process: 10 min (min) at 1000×g and 10°C to obtain cell supernatant and an additional centrifugation step with cell supernatant at 7000×g for 30 min at 10°C to remove cell debris. Supernatant was filtered through a 0.22 μm membrane filter and stored at 4°C until purification.

#### Antibody purification

Following transient transfection, antibodies were purified from cell supernatant by affinity chromatography using Protein A resin (MabSelect SuRe). Elution from the column was done with 50 mM citrate pH 3.2 and the eluate was adjusted to pH 7 by addition of Tris pH 9. The eluate was spun down and filtered through a 0.22 μm membrane filter and further purified by preparative size exclusion chromatography using the HiLoad Superdex 200 pg 26/60 column (Sigma-Aldrich). During size exclusion chromatography the buffer was exchanged to PBS pH 7 and the final material was filtered through a 0.22 μm membrane filter.

#### Biotinylation of SOSIP

Chemical biotinylation of the SOSIP Env trimer at primary amine groups was performed using a 10-fold molar excess of biotinylation reagent EZ-Link Sulfo-NHS-LC-Biotin (Thermo Fisher). Following overnight incubation of the biotinylation reagent with target protein at 2°C–8°C, buffer exchange to HBS-EP+ (Cytiva) was performed using three 0.5 mL Zeba spin desalting columns (7K MWCO, Thermo Fisher) consecutively. Biotinylated proteins were stored at ≤ −70°C.

#### SPR

SPR analyses were performed at Biaffin GmbH & Co KG, using a proprietary assay format. Briefly, modified sensor chips were coated with an avidin variant, followed by reversible immobilization of biotin-containing human FcRs (Acro Biosystems) or SOSIP Env trimer, which were injected at 2–5 μL/min until the aspired surface density was reached. Next, antibody analytes, purified with size exclusion chromatography, were serially diluted and injected at 30–50 μL/min in respective analysis buffers (10 mM HEPES (pH 7.4), 150 mM NaCl, 3 mM EDTA, 0.05% Tween 20 for FcγRs, SOSIP, and FcRn (pH 7.4) assays, or 50 mM Na-Acetate pH 6.0, 150 mM NaCl, 3 mM EDTA, 0.05% Tween 20 for FcRn (pH 6.0) assay). FcRs assays were performed at 25°C and SOSIP assay at 37°C. A flow cell containing avidin variant, but no biotinylated ligand served as reference. Analyses were performed in triplicate. Data processing and evaluation were performed by global fitting, applying a 1:1 binding model (FcγRI), a steady state approach (FcγRIIa (H167) and FcRn), a two-state reaction model (FcγRIIIa (V176)), or a bivalent analyte binding model (SOSIP), using Biacore T200 Evaluation Software version 3.2. Statistical evaluation of data (mean, SD and CV) was conducted using Excel (Microsoft).

#### SOSIP ELISA

BNT351 binding to the stabilized HIV-1 Env trimer (SOSIP_BG505.664_; ATUM, custom-made) was evaluated with ELISA using 96-well streptavidin plates (Nunc). Wells were coated with 100 ng/100 μL biotinylated recombinant SOSIP_BG505.664_. Plates were incubated at 4°C overnight, then washed with PBS-T (PBS with 0.01% Tween 20), and blocked with casein blocking buffer (Sigma-Aldrich) at 37°C for 1 h on a shaker. After another round of washing, serially diluted BNT351 in blocking buffer or negative control (blocking buffer alone) were added in duplicate and incubated at 37°C for 1 h on a shaker.

After washing the plates, horseradish peroxidase (HRP)-conjugated goat anti-human IgG secondary antibody was added (Jackson Immuno Research #109-035-098, 1:5000 in blocking buffer), and plates were incubated at 37°C for 45 min on a shaker. The plates were washed again, 3,3′,5,5′-tetramethylbenzidine (TMB) substrate (Biotrend Chemikalien GmbH) was added and incubated for 8 min at room temperature. The reaction was stopped with 100 μL 25% sulfuric acid and the absorbance (450 nm, ref. 620 nm) was measured on Epoch microplate reader (BioTek). Mean changes in optical density (ΔOD450-620 nm) were calculated.

#### Retrogenix cell microarray

The Retrogenix cell microarray^30^ was performed by Charles River Laboratories and included 6,105 full-length human proteins (plasma membrane proteins, secreted or a cell surface-tethered secreted proteins) and 400 human heterodimers. Briefly, expression vectors (pIRES-insert-IRES-ZsGreen1) encoding test proteins, CD20 (rituximab target), and EGFR (transfection and negative control) were individually arrayed in duplicate across cell microarray slides, followed by addition of HEK293 cells for reverse transfection and expression. After fixing the cells, slides were spotted with gelatin ±1.5 mg/mL CN54gp140 (Polymun #ENV002). Next, BNT351 (5 μg/mL) or the control antibody (rituximab biosimilar, Charles River 1 μg/mL) were added. Binding was detected with the Alexa Fluor 647 labeled anti-human IgG Fc. Fluorescent images were analyzed using ImageQuant software (GE healthcare, Version 8.2). Interactions were classified as “strong, medium, weak or very weak”, depending on the intensity of the duplicate spots. The assay included two replicate slide sets (each with duplicate spots).

#### Flow cytometry validation of Retrogenix findings

HEK293 cells were transfected with expression vectors encoding ZsGreen1 only, or ZsGreen1 and PGC or FCGR3A + FCER1G. Non-transfected cells served as a negative control. After incubating live transfectant cells or non-transfected cells with 5 μg/mL BNT351, they were washed and incubated with the same AF647 anti-hIgG Fc detection antibody as used in the cell microarray screens. Cells were again washed and analyzed by flow cytometry. A 7AAD live/dead dye was used to exclude dead cells, and ZsGreen+ (transfected) cells were selected for analysis.

#### Pseudovirus production

Pseudoviruses were produced as previously described^31^ by co-transfecting HEK293T cells (50–80% confluent) with *Env*-expression plasmid or SGS-derived PCR products and Env-deficient HIV-1 backbone vector (pSG3Δenv), using FuGENE 6 reagent (Promega) according to the manufacturer’s instructions. Briefly, the transfection complexes were incubated for 30 min at room temperature (18°C–25°C), after which they were added to HEK293T cells and incubated for 48 to 72 h with one change of medium after the first 3 to 8 h. Virus-containing culture fluid was removed from the flasks and filtered through a 0.45 μm filter to eliminate cell debris. For pseudoviruses produced using *Env*-expression plasmids, FBS was added to a final concentration of 20% before storing the virus-containing culture fluid at ≤ -70°C.

For SGS-derived PCR fragments, an overlap PCR product encompassing the cytomegalovirus (CMV) promoter and an env/rev cassette was produced as described previously.^32^ In brief, a CMV promoter PCR product was amplified from an expression plasmid, and env/rev cassettes were amplified from the first-round SGS PCR product using primers env1Atopo CACCGGCTTAGGCATCTCCTATGGCAGGAAGAA and envN CTGCCAATCAGGGAAGTAGCCTTGTGT. CMV promoter and env/rev overlap PCR was performed using primers CMVenv AGTAATCAATTACGGGGTCATTAGTTCAT and Rev19 ACTTTTTGACCACTTGCCACCCAT. Env/rev and overlap PCRs were performed using Phusion Hot Start Flex DNA Polymerase (New England Biolabs).

#### TZM-bl pseudovirus neutralization assay

TZM-bl neutralization assay was performed as previously described.^31^ Briefly, pseudoviruses (titrated to produce a luminescence signal of approximately 100,000 relative light units (RLUs) or at least 10-fold above background) and dilution series of antibodies or HIV-1 entry inhibitors—maraviroc (NIH AIDS Reagent program), ibalizumab (kind gift from Dr. David Ho at Aaron Diamond AIDS Research Center, NY), and fostemsavir (Sigma-Aldrich)—were mixed and co-incubated at 37°C for 1 h, followed by addition of TZM-bl cells (10^4^ cells per well) on a 96-well plate in 250 μL medium supplemented with 10–11 μg/mL DEAE-dextran. Wells containing cells and pseudovirus (without sample) or cells alone served as positive and negative infection controls, respectively. Following a 2-day incubation at 37°C and 5% CO_2_, the luminescence was detected with a luminometer after adding Bright-Glo luciferase reagent (Promega) or lyse/luc buffer (10 mM MgCl_2_, 0.3 mM ATP, 0.5 mM Coenzyme A, 17 mM IGEPAL (all Sigma-Aldrich), and 1 mM D-Luciferin (GoldBio) in Tris-HCL. After subtracting background RLUs of non-infected TZM-bl cells, IC_50_ and IC_80_ values were determined as the antibody concentration resulting in a 50% or 80% RLU reduction, respectively, compared to untreated virus control wells. Geometrical mean IC_50_ and IC_80_ values were calculated including all tested strains. For resistant strains, upper neutralization threshold was used for calculation: 50 μg/mL for BNT351 and 25 μg/mL for 1–18 for multiclade panel, 10 μg/mL for AMP panel, and 25 μg/mL for tropism panel.

#### Oxidation of tryptophan and methionine residues and tryptic digestion

To test the oxidation of tryptophan and methionine residues, the BNT351 antibody buffer was exchanged for 20 mM sodium citrate, 140 mM NaCl, pH 5.5 (methionine oxidation) or 20 mM sodium citrate, 140 mM NaCl, 260 mM L-methionine, pH 5.5 (tryptophan oxidation), using Amicon Ultra centrifuge filter with a cutoff of 10 kDa. The sample was then incubated with 2 mM H_2_O_2_ for 4.5 h at 2°C–8°C (methionine oxidation) or with 0.5% AAPH for 5 days at 40 °C (tryptophan oxidation). An untreated aliquot served as a reference (unstressed sample). Afterward the antibody samples were denatured and reduced with denaturing buffer (0.4 M Tris/HCl, 8.0 M Guanidine-HCl, pH 8.0) and freshly prepared DTT solution in denaturing buffer at 37°C for 1 h (550 rpm). The reduced samples were cooled to room temperature and subsequently alkylated for 15 min at room temperature in the dark by addition of iodoacetamide. The excess of alkylation reagent was inactivated by the addition of DTT solution. The samples were then buffer-exchanged to 50 mM Tris/HCl, 100 mM L-methionine, pH 7.5 using Sephadex G-25 DNA grade NAP-5 columns (Illustra 17-0853-01). Digestion was performed with trypsin (Promega, #V528A) for 20 h at 37°C at an enzyme to substrate ratio of 1:50 (w/w). The digestion was stopped by addition of 10% trifluoroacetic acid (ULC-MS-Ortigrade LGC). The digested samples were stored at ≤ −20°C until analysis by tandem mass spectrometry.

#### Isomerization and succinimide formation and tryptic digestion

To test the modification of asparagine and aspartate residues under stress conditions, the BNT351 buffer was exchanged for 20 mM histidine, 140 mM NaCl, pH 6.0 using AmiconUltra 0.5 centrifuge filter with a cutoff of 10 kDa. The sample was then incubated at 40°C for 2 or 4 weeks. An untreated aliquot served as a reference (unstressed sample).

Afterward samples were denatured and reduced with denaturing buffer (0.2 M His/NaCl, 8.0 M Guanidine-HCl, pH 6.0) and freshly prepared 10 mM TCEP solution in denaturing buffer at 37°C for 1 h. The reduced samples were cooled to room temperature. No alkylation was performed. The samples were then buffer-exchanged to 0.2 M His/NaCl, 0.5 mM TCEP-HCl, 50 mM L-methionine pH 6.0 using Sephadex G-25 DNA grade NAP-5 columns (Illustra #17-0853-01). Digestion was performed with trypsin/LysC (Promega, #V5073) for 20 h at 37°C at an enzyme to substrate ratio of 1:50 (w/w). The digestion was stopped by the addition of 20 μL 10% trifluoro acetic acid. The digested samples were stored at ≤ -20°C until analysis by tandem mass spectrometry.

#### On-line reversed phase chromatography–tandem mass spectrometry (LC-MS/MS)

The peptides in the digested samples were separated using reversed phase HPLC (RSLC3000, Thermo Scientific Dionex) and a capLC column (Waters, Acquity Peptide BEH C18, #186005594, 300 Å, 1.7 μm, 1 mm × 150 mm). An optimized 90-min linear gradient (eluent A: H_2_O + 0.1% formic acid, eluent B: acetonitrile with 0.1% formic acid) was applied at 55°C. The HPLC eluate was directly infused into an Eclipse mass spectrometer (Thermo scientific). The mass spectrometer operated in positive ion mode, the spray voltage was 3.5 kV, the capillary temperature was 250°C and vaporizer temperature 75°C. For the MS/MS product ion scan, the activation type was higher energy CID (HCD). The 4-scan-event method applied consisted of a full MS survey scan at m/z 200-2,000 and resolution power (RP) of 120,000 followed by three cycles of data-dependent MS/MS scans on the top three most intense ions. The dynamic exclusion function was enabled, and parameters were as follows: dynamic exclusion of 90 s (s), isolation window is set automatically, resolution of 30,000, normalized AGC target 2,000% (MS/MS), maximum inject time Auto, HCD collision energy of 25%, intensity threshold of 5×10^4^. Unassigned charge states and charge state +1 and ≥8 were rejected for MS/MS triggering.

#### Mouse PK study

Tg32 mice were administered a single IV bolus dose of BNT351 (5 mg/kg or 30 mg/kg) at the retro-orbital sinus. Serial serum microsamples were collected at various timepoints from the tail vein and stored at −70°C until measured using a fit-for-purpose sandwich immunoassay (MSD) to determine the amount of BNT351 in the serum. A control group was not considered necessary for this experiment.

#### Cynomolgus macaques PK study

Cynomolgus macaques were administered with 5 mg/kg or 45 mg/kg BNT351 by IV bolus injections in a saphenous or cephalic vein. Serial serum samples were collected at selected time points and stored at −70°C until measurement. Serum concentrations were measured by a fit-for-purpose sandwich immunoassay (MSD) to determine the amount of BNT351 in the serum. A control group was not considered necessary for this experiment.

#### Sandwich immunoassay (MSD)

Mouse- and monkey-specific fit-for-purpose qualified sandwich immunoassays were established, in which a custom-made biotinylated anti-idiotypic (anti-1-18 F(ab')_2_) capture antibody (Bio Rad AbD Serotec, GmbH) was immobilized (0.25 μg/mL) in a 96-well streptavidin-coated plate (MSD). The analyte bound to the capture antibody. A Ruthenium-labeled anti-human IgG detection antibody (AffiniPure Goat Anti-Human IgG (Jackson ImmunoResearch #109-005-098), internally labeled with Sulfo-Tag) was used at 0.05 μg/mL to generate an electrochemiluminescent response that was directly proportional to the amount of analyte detected in the well. Back-calculated concentration of analyte was generated using a 4 PL fit model with 1/Y^2^ weighting.

#### Non-compartmental analysis (NCA)

A non-compartmental analysis (NCA) of BNT351 in serum of mice and cynomolgus macaques was performed (Phoenix NLME software version 8.3.5.340). AUC was calculated using the Linear Up Log Down method with extrapolated C_0_. AUC_inf_ (area under the concentration-time curve extrapolated to infinity) was extrapolated using the predicted Clast/λz. PK profile from a cynomolgus macaque that showed a sharp drop in concentration, typical of ADA interference, were excluded from calculations of mean values.

#### Two-compartmental population PK model

The population PK analysis for mouse or cynomolgus macaque serum concentrations was performed using Phoenix NLME software (version 8.3.5.340) with the first-order conditional estimation extended-least-squares method (FOCE-ELS) to determine typical values for clearance (CL), intercompartmental clearance (Q), volume of distribution of the central compartment (Vc) and volume of distribution of peripheral compartment (Vp). Dataset preparation was performed using Excel (Microsoft).

#### Allometric scaling

Interspecies scaling was used for the prediction of human PK from preclinical data. Allometric scaling of human PK parameters was performed in Microsoft Excel according to the following formula:

$${CL}_{human}={CL}_{animal}\times {\left(\frac{{BW}_{human}}{{BW}_{animal}}\right)}^{b_{CL}}$$

$$Q_{human}=Q_{animal}\times {\left(\frac{{BW}_{human}}{{BW}_{animal}}\right)}^{b_{Q}}$$

$$V_{c,human}=V_{c,animal}\times {\left(\frac{{BW}_{human}}{{BW}_{animal}}\right)}^{b_{Vc}}V_{p,human}=V_{p,animal}\times {\left(\frac{{BW}_{human}}{{BW}_{animal}}\right)}^{b_{Vp}}$$

Abbreviations: b = Allometric exponent; BW = bodyweight; V_c_ = volume of distribution in the central compartment; V_p_ = volume of distribution in the peripheral compartment; CL = clearance from the central compartment; Q = intercompartmental clearance between central and peripheral compartment.

For Fc-engineered antibodies the typical allometric exponents should be adapted to reflect the increased FcRn binding affinity. Therefore, published optimized exponents for Fc-engineered antibodies were used for scaling Tg32 mice^13^ and cynomolgus macaques^14^ PK parameters to human, as specified in the table below.

**Table:** Allometric scaling exponents (b) for Fc-engineered mAbs

Parameter	Exponent Tg32 mouse	Exponent cynomolgus macaque
V_c_	0.95	0.95
V_p_	0.87	0.95
CL	0.73	0.55
Q	0.6	0.6

Fc, fragment crystallizable; mAb, monoclonal antibody; V_c_, volume of distribution in the central compartment; V_p_, volume of distribution in the peripheral compartment; CL, clearance from the central compartment; Q, intercompartmental clearance between central and peripheral compartment.

#### Recombinant HIV-1 production

Replication-competent recombinant HIV-1 (YU2 Env in NL4-3 backbone^29^) was produced by transfection of HEK293T cells using FuGENE 6 transfection reagent (Promega). Harvested viral supernatants were stored at −80°C to −150°C.

#### HIV-1 infection and animal treatment in humanized CD34^+^ NSG mice

Mice were challenged twice on two consecutive days with replication-competent HIV-1 intraperitoneally. For each challenge, 100 μL of pure viral supernatant was used. For the treatment study, HIV-1-infected mice with confirmed viremia were started on SC treatment with BNT351 4 weeks after the HIV-1 challenge. Following a 1 mg loading dose, a dose of 0.5 mg was injected every 3–4 days for 8 weeks. The control group was given DPBS SC injections on the same days. All SC injections were given in the neck scruff in a 250 μL dose volume. For the PK study, one dose of 0.5 mg BNT351 was given IV 7 weeks after the HIV-1 challenge and confirmation of viremia by qRT-PCR. Non-infected humanized CD34^+^ NSG mice served as control.

#### Gyros ELISA for BNT351 quantification

Gyros ELISA analyses were conducted to quantify BNT351 levels in NSG mouse serum using kits, microtiter assay plates, a measuring device, and analysis software from Gyros Protein Technologies AB. For capture, 0.1 mg/mL of a custom-made biotinylated anti-idiotypic (anti-1-18 F(ab')_2_) antibody was used (BioRad AbD Serotec, GmbH). Detection was performed using 25 nM of an Alexa Fluor 647 labeled F(ab')_2_ Fragment Rabbit Anti-Human IgG, Fcγ (Jackson ImmunoResearch, #309-606-008). The capture antibody was diluted in 1× PBS-Tween 20 Buffer (Thermo Fisher Scientific), while the detection antibody was diluted in Rexxip F buffer. The assay was processed in a Gyrolab Bioaffy 20 HC CD.

Serum samples diluted 1:2 in DPBS were inactivated in by incubating in Triton X-100 at a final concentration of 1%. For sample preparation, the Reagent F buffer (Gyrolab 2G Generic PK Type F Kit Reagents) was used to dilute serum samples 10-fold. The CD columns were washed with Reagent C and Reagent D (Gyrolab 2G Generic PK Type F Kit Reagents). An eleven-point, 3-fold serial dilution of the 1–18 IgG reference protein diluted in 10% NSG serum was used to calculate BNT351 concentrations. The working range for determining BNT351 concentrations in NSG serum was 12.3–27000 ng/mL. All data were generated on a Gyros xPand ELISA device, and results analyzed with Gyrolab Evaluator software.

#### Viral load measurements in humanized CD34^+^ NSG mice

Blood was collected from the facial vein into EDTA tubes on indicated days for plasma isolation. Plasma RNA was extracted using the MinElute Virus Mini Spin Kit (QIAGEN), including an on-column DNase I (QIAGEN) digestion step. Viral loads were determined by RT-qPCR performed on a QuantStudio 5 (Thermo Fisher) using a *pol*-specific primer/probe mix and TaqMan RNA-to-CT 1-Step-Kit (Thermo Fisher). Per reaction, 20 μL purified plasma RNA and 30 μL primer/probe mix (5′-TAATGGCAGCAATTTCACCA primer, 5′-GAATGCCAAATTCCTGCTTGA primer,/56-FAM/CCCACCAACARGCRGCCTTAACTG/ZenDQ/probe, (all from IDT) were diluted in nuclease-free water), mixed and pipetted into individual wells of a white PCR plate. An SupT1-R5 cell infection-derived HIV-1_YU2_ standard with known HIV-1 RNA copy number determined using the quantitative cobas 6800 HIV-1 kit (Roche) was included for every PCR run (1:5 dilution series in AVE buffer (QIAGEN)). RT-qPCR conditions were: 48°C for 15 min, 95°C for 10 min, and 45 cycles of 95°C/15 s and 60°C/1 min. The limit of accuracy of the qRT-PCR was determined as 774 copies/mL.

#### Plasma single genome sequencing

SGS was performed as described before with modifications.^3^ Briefly, cDNA was generated from isolated plasma RNA using the antisense primer YB383 5′-TTTTTTTTTTTTTTTTTTTTTTTTRAAGCAC and Superscript IV reverse transcriptase (Thermo Fisher) according to the manufacturer’s protocol, followed by incubation with 0.25 U/μL RNase H (Thermo Fisher) at 37°C for 20 min. Env cDNA was subsequently amplified by nested PCR: first-round PCR was conducted using primers YB383 5′-TTTTTTTTTTTTTTTTTTTTTTTTRAAGCAC and YB50 5′-GGCTTAGGCATCTCCTATGGCAGGAAGA, and run at 94°C for 2 min; 35 cycles of 94°C for 30 s, 55°C for 30 s, and 72°C for 4 min; and 72°C for 15 min 1 μL first-round PCR product was then used as template for the second-round PCR that was conducted using primers YB49 5′-TAGAAAGAGCAGAAGACAGTGGCAATGA and YB52 5′-GGTGTGTAGTTCTGCCAATCAGGGAAGWAGCCTTGTG, and run at 94°C for 2 min; 45 cycles of 94°C for 30 s, 55°C for 30 s, and 72°C for 4 min; and 72°C for 15 min. PCR was performed using the Platinum Taq Green Hot Start DNA Polymerase (Thermo Fisher). All primers were from IDT. PCR products were sequenced by Sanger sequencing and only PCR products without sequence ambiguities were included in the analysis.
