# Supplementary material for: Preclinical assessment of broadly neutralizing HIV-1 antibody BNT351 with optimized pharmacokinetics and potent antiviral activity
Source: iScience. 2026 Jun 11;29(6):116022. doi: 10.1016/j.isci.2026.116022 (PMC13276592; doi:10.1016/j.isci.2026.116022)
Supplement: Document S1. Figures S1–S6 and Tables S1–S5 [file mmc1.pdf]

iScience, Volume *29*

Supplemental information

Preclinical assessment of broadly neutralizing HIV-1 antibody BNT351 with optimized pharmacokinetics and potent antiviral activity

Sven Kratochvil, Maximilian Kullmann, Henning Gruell, Sophie Sayettat, Chia-Hung Tsai, Natasa Vukovic, Christine Janaitis, Claudia Lindemann, Sandra Praßl, Ricarda Stumpf, Jacqueline Knüfer, Felix Tolksdorf, Uğur Şahin, Johannes Nelke, Alexandra Malz, Philipp Schommers, Sinethemba Bhebhe, Nonhlanhla Mkhize, Penny Moore, Michael S. Seaman, Florian Klein, and Valentin Le Douce

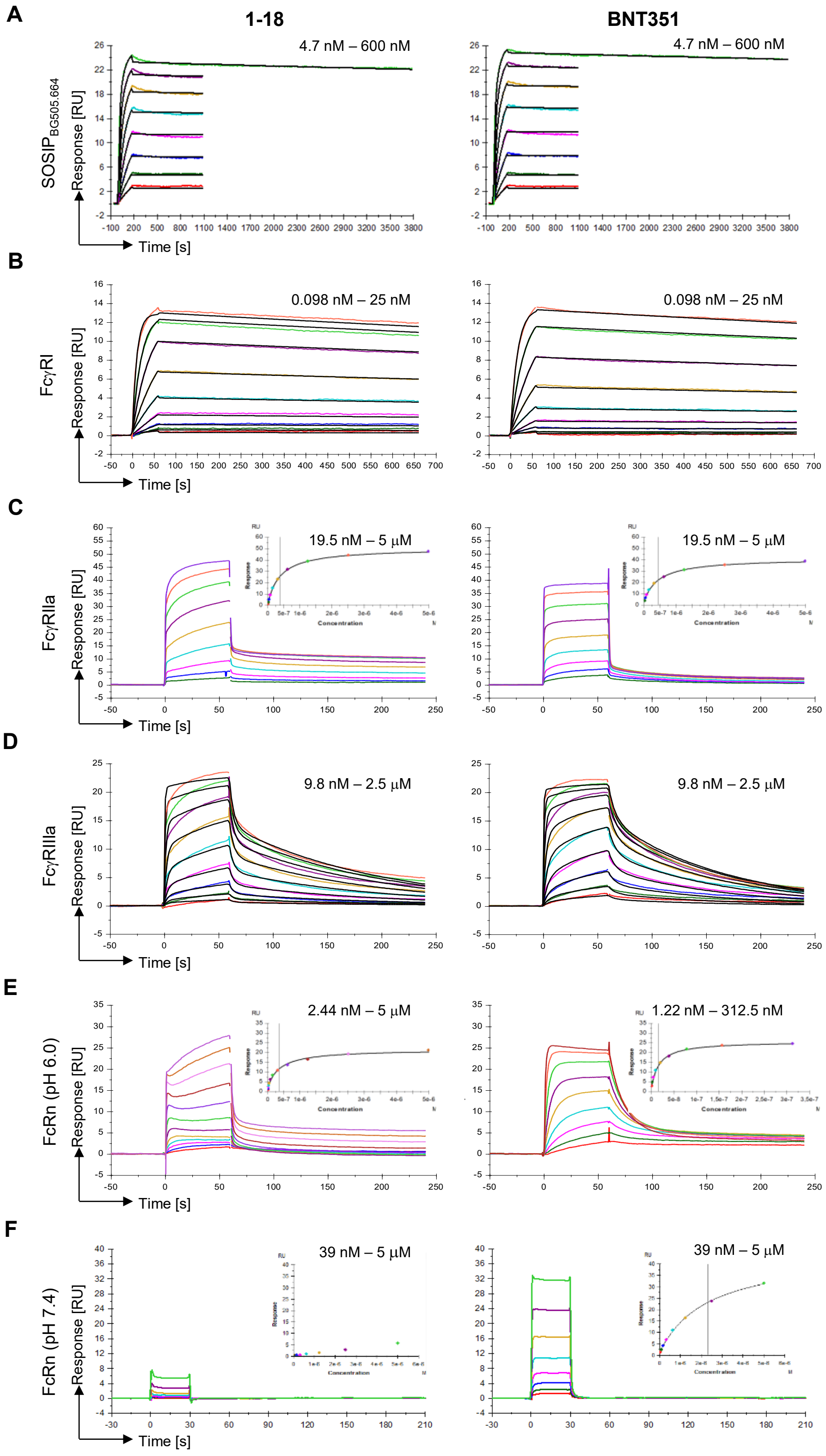

Figure S1. Representative sensorgrams of 1-18 and BNT351 interacting with human HIV-1 Env trimer and Fc receptors, related to Figure 1A, Figure 4A, and Figure S4

1-18 and BNT351 binding to immobilized HIV-1 Env trimer (SOSIP$_{BG505.664}$) and Fc receptors was analyzed via SPR. 1-18 and BNT351 were injected in serial two-fold dilutions, with concentrations indicated on each graph. The recorded data (colored curves) were fitted applying (A) the bivalent analyte binding model (black curves), (B) 1:1 binding model (black curves), (D) the two-state reaction model (black curves), or (C, E, F) steady state approach (saturation curves shown as inserts).

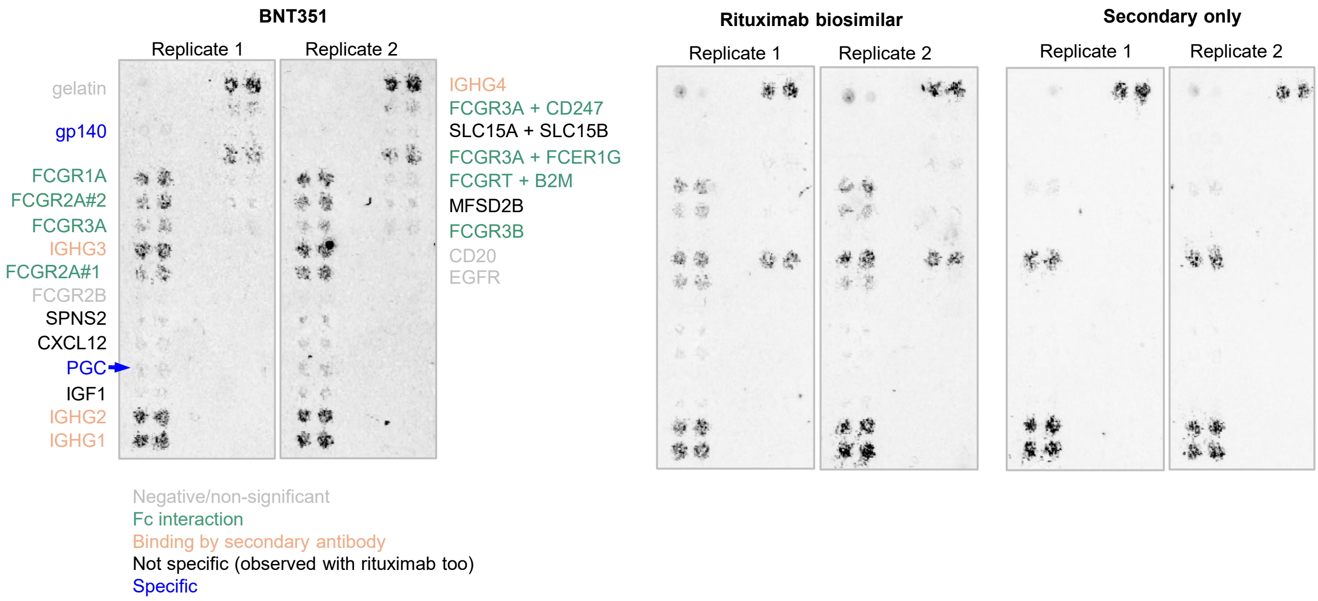

Figure S2. Dotblots of the Retrogenix® assay used for in vitro off-target profiling of BNT351, related to Figure 1B

BNT351's off-target binding was assessed with Retrogenix® Cell Microarray Technology platform. Interactions identified during the library screen with ~6,500 human proteins are shown. Rituximab biosimilar and secondary only antibody were used as controls.

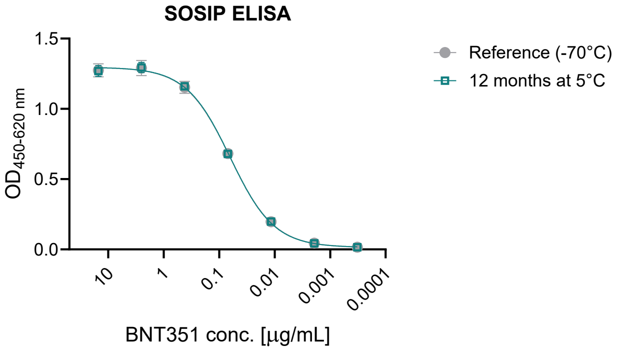

Figure S3. No loss of binding potency under normal storage conditions for 12 months, related to Figure 3

Binding of BNT351 stored at 5±3ºC for 12 months to SOSIPB$_{G505.664}$ by ELISA (mean ± standard deviation of quadruplicate measurements). For reference, BNT351 stored at -70±10ºC was thawed and used. Curves were fitted using 4PL sigmoidal model.

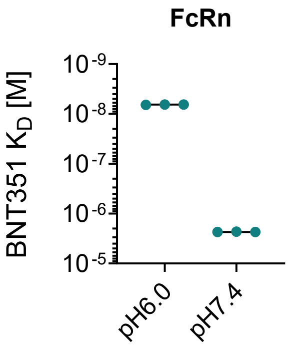

Figure S4. BNT351 affinity to human FcRn is increased only at pH 6.0, related to Figure 4A

The binding affinity of BNT351 to human FcRn by surface plasmon resonance (SPR) at indicated pH values. Analysis was performed in triplicate; mean and individual values are shown.

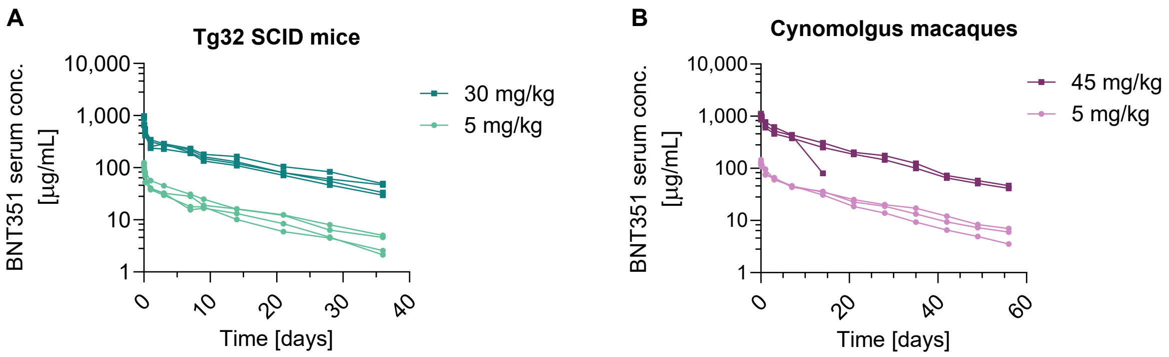

Figure S5. BNT351 pharmacokinetic data of individual animals, related to Figure 4B

(A) BNT351 serum concentration in Tg32 SCID mice (n=4) injected intravenously (IV) with a single dose of 5 mg/kg or 30 mg/kg antibody on Day 0. (B) BNT351 serum concentration in cynomolgus macaques (n=3) injected IV with a single dose of 5 mg/kg or 45 mg/kg antibody on Day 0. Data of individual animals are shown in both panels.

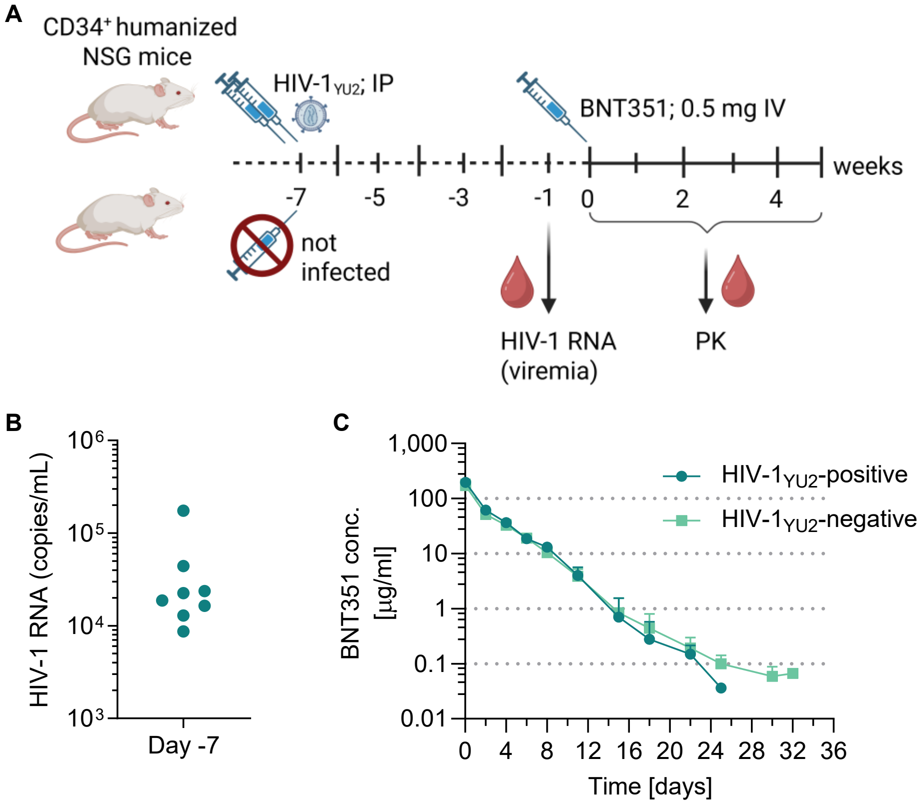

Figure S6. BNT351 serum concentration is not dependent on HIV-1 presence in mice, related to Figure 4

CD34⁺ humanized NSG mice were challenged intraperitoneally (IP) with HIV-1_{YU2} on Day -49 and Day -50. Mice with confirmed viremia on Day -7 (n=8) and non-infected mice (n=8) received a single intravenous (IV) dose of 0.5 mg BNT351 on Day 0. (A) Experiment schema. (B) HIV-1 RNA plasma copies of HIV-1_{YU2}-infected mice on Day -7. Values of individual mice are shown. (C) BNT351 serum concentration was determined via Gyros ELISA using anti-idiotypic detection antibody. Mean values ± SD are shown. PK = pharmacokinetics

Table S1. IC$_{50}$ values of 1-18 and BNT351 against the multiclade panel, related to Figure 2A

HIV-1 strain	Clade	IC$_{50}$ 1-18	IC$_{50}$ BNT351
6535.3	B	0.068	0.07
QH0692.42	B	0.089	0.088
SC422661.8	B	0.009	0.007
PVO.4	B	0.017	0.036
TRO.11	B	0.015	0.01
AC10.0.29	B	0.369	0.8
RHPA4259.7	B	0.015	0.035
THRO4156.18	B	1.584	3.245
REJO4541.67	B	0.016	0.011
TRJO4551.58	B	0.06	0.068
WITO4160.33	B	0.006	0.02
CAAN5342.A2	B	0.804	0.61
WEAU_d15_410_787	B (T/F)	0.01	0.029
1006_11_C3_1601	B (T/F)	0.039	0.033
1054_07_TC4_1499	B (T/F)	0.475	0.228
1056_10_TA11_1826	B (T/F)	0.033	0.041
1012_11_TC21_3257	B (T/F)	0.008	0.011
6240_08_TA5_4622	B (T/F)	0.312	0.175
6244_13_B5_4576	B (T/F)	0.023	0.031
62357_14_D3_4589	B (T/F)	0.06	0.084
SC05_8C11_2344	B (T/F)	0.045	0.019
Du156.12	C	0.022	0.015
Du172.17	C	>25	24.223
Du422.1	C	0.063	0.04
ZM197M.PB7	C	0.091	0.085
ZM214M.PL15	C	0.048	0.083
ZM233M.PB6	C	0.042	0.038
ZM249M.PL1	C	0.02	0.011
ZM53M.PB12	C	0.256	0.247
ZM109F.PB4	C	9.916	7.468
ZM135M.PL10a	C	1.729	2.21
CAP45.2.00.G3	C	>25	>50
CAP210.2.00.E8	C	0.818	0.939
HIV-001428-2.42	C	0.007	0.005
HIV-0013095-2.11	C	0.151	0.059
HIV-16055-2.3	C	0.019	0.017
HIV-16845-2.22	C	0.862	0.448
Ce1086_B2	C (T/F)	0.092	0.039
Ce0393_C3	C (T/F)	0.036	0.178
Ce1176_A3	C (T/F)	0.199	0.14
**Controls**			
YU2	B	0.032	0.027
BG505.T332N	A	0.054	0.053

HIV-1 strain	Clade	IC$_{50}$ 1-18	IC$_{50}$ BNT351
Ce2010_F5	C (T/F)	0.139	0.12
Ce0682_E4	C (T/F)	0.015	0.012
Ce1172_H1	C (T/F)	0.295	0.199
Ce2060_G9	C (T/F)	0.086	0.1
Ce703010054_2A2	C (T/F)	0.032	0.019
BF1266.431a	C (T/F)	0.03	0.02
246F C1G	C (T/F)	0.006	0.009
249M B10	C (T/F)	0.027	0.104
ZM247v1(Rev-)	C (T/F)	0.023	0.015
7030102001E5(Rev-)	C (T/F)	0.121	0.053
1394C9G1(Rev-)	C (T/F)	0.287	0.356
Ce704809221_1B3	C (T/F)	0.078	0.07
CNE19	BC	0.006	0.01
CNE20	BC	0.033	0.03
CNE21	BC	0.039	0.065
CNE17	BC	0.157	0.202
CNE30	BC	0.076	0.117
CNE52	BC	0.007	0.01
CNE53	BC	0.089	0.075
CNE58	BC	0.017	0.028
MS208.A1	A1D	0.031	0.022
Q23.17	A1	0.017	0.018
Q461.e2	A1	0.053	0.03
Q769.d22	A1	0.02	0.009
Q259.d2.17	A1	0.018	0.022
Q842.d12	A1	0.006	0.006
0260.v5.c36	A1	0.076	0.048
3415.v1.c1	A1	0.01	0.011
3365.v2.c20	A1	0.025	0.045
191955_A11	A (T/F)	0.017	0.015
191084 B7-19	A (T/F)	0.023	0.037
9004SS_A3_4	A (T/F)	0.031	0.026
T257-31_CRF02	AG	0.05	0.081
928-28_CRF02	AG	0.189	0.206
263-8_CRF02	AG	0.016	0.024
T250-4_CRF02	AG	0.019	0.024
T251-18_CRF02	AG	0.044	0.047
T278-50_CRF02	AG	>25	32.017
T255-34_CRF02	AG	0.042	0.015
211-9_CRF02	AG	0.076	0.17

HIV-1 strain	Clade	IC$_{50}$ 1-18	IC$_{50}$ BNT351
235-47_CRF02	AG	0.007	0.002
620345.c01_CRF01	AE	0.145	0.14
CNE8_CRF01	AE	0.005	0.013
C1080.c03_CRF01	AE	0.02	0.048
R2184.c04_CRF01	AE	0.004	0.006
R1166.c01_CRF01	AE	0.006	0.012
R3265.c06_CRF01	AE	0.01	0.027
C2101.c01_CRF01	AE	0.038	0.04
C3347.c11_CRF01	AE	0.027	0.015
C4118.c09_CRF01	AE	0.04	0.028
CNE5_CRF01	AE	0.184	0.129
BJOX009000.02.4	AE	0.386	0.254
BJOX015000.11.5_CRF01	AE (T/F)	0.117	0.106
BJOX010000.06.2_CRF01	AE (T/F)	0.526	0.26
BJOX025000.01.1_CRF01	AE (T/F)	0.12	0.052
BJOX028000.10.3_CRF01	AE (T/F)	0.806	0.136
X1193_c1	G	0.05	0.017
P0402_c2_11	G	0.043	0.005
X1254_c3	G	0.027	0.046
X2088_c9	G	0.089	0.043
X2131_C1_B5	G	0.053	0.048
P1981_C5_3	G	11.131	7.614
X1632_S2_B10	G	0.017	0.017
3016.v5.c45	D	1.227	0.709
A07412M1.vrc12	D	0.043	0.041
231965.c01	D	0.115	0.093
231966.c02	D	0.096	0.05
6405.v4.c34	D	>25	>50
3817.v2.c59	CD	0.131	0.181
6480.v4.c25	CD	0.023	0.013
6952.v1.c20	CD	0.035	0.069
6811.v7.c18	CD	0.042	0.048
89-F1_2_25	CD	0.007	0.011
3301.v1.c24	AC	0.005	0.013
6041.v3.c23	AC	0.004	0.01
6540.v4.c1	AC	0.005	0.014
6545.v4.c1	AC	0.004	0.01
0815.v3.c3	ACD	0.009	0.019
3103.v3.c10	ACD	0.05	0.057
GeoMean		0.048	0.053

Color code (µg/mL):	<0.1	0.1 – 0.5	0.5 – 1	>1

Table S2. IC$_{80}$ values of 1-18 and BNT351 against the multiclade panel, related to Figure 2A

HIV-1 strain	Clade	IC$_{80}$ 1-18	IC$_{80}$ BNT351
6535.3	B	0.353	0.506
QH0692.42	B	0.397	0.311
SC422661.8	B	0.034	0.030
PVO.4	B	0.059	0.113
TRO.11	B	0.054	0.053
AC10.0.29	B	1.772	2.774
RHPA4259.7	B	0.066	0.111
THRO4156.18	B	9.632	17.384
REJO4541.67	B	0.059	0.078
TRJO4551.58	B	0.237	0.244
WITO4160.33	B	0.033	0.058
CAAN5342.A2	B	3.427	3.027
WEAU_d15_410_787	B (T/F)	0.059	0.088
1006_11_C3_1601	B (T/F)	0.146	0.158
1054_07_TC4_1499	B (T/F)	1.656	1.437
1056_10_TA11_1826	B (T/F)	0.192	0.201
1012_11_TC21_3257	B (T/F)	0.042	0.034
6240_08_TA5_4622	B (T/F)	1.073	1.071
6244_13_B5_4576	B (T/F)	0.148	0.093
62357_14_D3_4589	B (T/F)	0.258	0.258
SC05_8C11_2344	B (T/F)	0.159	0.058
Du156.12	C	0.081	0.062
Du172.17	C	>25	>50
Du422.1	C	0.216	0.227
ZM197M.PB7	C	0.321	0.445
ZM214M.PL15	C	0.654	0.534
ZM233M.PB6	C	0.159	0.140
ZM249M.PL1	C	0.073	0.068
ZM53M.PB12	C	1.204	0.957
ZM109F.PB4	C	>25	>50
ZM135M.PL10a	C	13.124	14.169
CAP45.2.00.G3	C	>25	>50
CAP210.2.00.E8	C	4.420	4.193
HIV-001428-2.42	C	0.019	0.018
HIV-0013095-2.11	C	0.534	0.284
HIV-16055-2.3	C	0.051	0.050
HIV-16845-2.22	C	2.869	3.506
Ce1086_B2	C (T/F)	0.238	0.191
Ce0393_C3	C (T/F)	0.162	0.947
Ce1176_A3	C (T/F)	0.694	0.680
**Controls**			
YU2	B	0.103	0.101
BG505.T332N	A	0.168	0.173

HIV-1 strain	Clade	IC$_{80}$ 1-18	IC$_{80}$ BNT351
Ce2010_F5	C (T/F)	0.477	0.478
Ce0682_E4	C (T/F)	0.071	0.048
Ce1172_H1	C (T/F)	1.051	0.719
Ce2060_G9	C (T/F)	0.408	0.411
Ce703010054_2A2	C (T/F)	0.162	0.122
BF1266.431a	C (T/F)	0.115	0.081
246F C1G	C (T/F)	0.021	0.026
249M B10	C (T/F)	0.099	0.824
ZM247v1(Rev-)	C (T/F)	0.106	0.082
7030102001E5(Rev-)	C (T/F)	0.417	0.272
1394C9G1(Rev-)	C (T/F)	1.296	1.744
Ce704809221_1B3	C (T/F)	0.362	0.327
CNE19	BC	0.022	0.038
CNE20	BC	0.149	0.104
CNE21	BC	0.185	0.224
CNE17	BC	0.566	0.707
CNE30	BC	0.274	0.413
CNE52	BC	0.026	0.030
CNE53	BC	0.317	0.219
CNE58	BC	0.050	0.067
MS208.A1	A1D	0.116	0.090
Q23.17	A1	0.062	0.047
Q461.e2	A1	0.200	0.089
Q769.d22	A1	0.088	0.056
Q259.d2.17	A1	0.066	0.067
Q842.d12	A1	0.014	0.021
0260.v5.c36	A1	0.238	0.145
3415.v1.c1	A1	0.042	0.033
3365.v2.c20	A1	0.105	0.147
191955_A11	A (T/F)	0.068	0.038
191084 B7-19	A (T/F)	0.085	0.109
9004SS_A3_4	A (T/F)	0.113	0.098
T257-31_CRF02	AG	0.199	0.289
928-28_CRF02	AG	0.672	0.690
263-8_CRF02	AG	0.058	0.067
T250-4_CRF02	AG	0.067	0.087
T251-18_CRF02	AG	0.137	0.135
T278-50_CRF02	AG	>25	>50
T255-34_CRF02	AG	0.292	0.109
211-9_CRF02	AG	0.283	0.489

HIV-1 strain	Clade	IC$_{80}$ 1-18	IC$_{80}$ BNT351
235-47_CRF02	AG	0.027	0.012
620345.c01_CRF01	AE	1.964	1.127
CNE8_CRF01	AE	0.030	0.066
C1080.c03_CRF01	AE	0.100	0.249
R2184.c04_CRF01	AE	0.020	0.030
R1166.c01_CRF01	AE	0.026	0.039
R3265.c06_CRF01	AE	0.086	0.205
C2101.c01_CRF01	AE	0.330	0.357
C3347.c11_CRF01	AE	0.090	0.109
C4118.c09_CRF01	AE	0.249	0.120
CNE5_CRF01	AE	0.868	0.447
BJOX009000.02.4	AE	1.300	1.944
BJOX015000.11.5_CRF01	AE (T/F)	0.773	0.703
BJOX010000.06.2_CRF01	AE (T/F)	3.408	1.841
BJOX025000.01.1_CRF01	AE (T/F)	0.839	0.298
BJOX028000.10.3_CRF01	AE (T/F)	>25	5.751
X1193_c1	G	0.151	0.051
P0402_c2_11	G	0.100	0.026
X1254_c3	G	0.126	0.144
X2088_c9	G	0.319	0.187
X2131_C1_B5	G	0.236	0.186
P1981_C5_3	G	>25	>50
X1632_S2_B10	G	0.084	0.078
3016.v5.c45	D	7.388	3.720
A07412M1.vrc12	D	0.190	0.203
231965.c01	D	0.515	0.311
231966.c02	D	0.495	0.191
6405.v4.c34	D	>25	>50
3817.v2.c59	CD	0.540	0.605
6480.v4.c25	CD	0.083	0.047
6952.v1.c20	CD	0.182	0.225
6811.v7.c18	CD	0.227	0.167
89-F1_2_25	CD	0.023	0.030
3301.v1.c24	AC	0.023	0.036
6041.v3.c23	AC	0.018	0.030
6540.v4.c1	AC	0.040	0.040
6545.v4.c1	AC	0.020	0.030
0815.v3.c3	ACD	0.044	0.050
3103.v3.c10	ACD	0.144	0.142
GeoMean		0.277	0.294

Color code (µg/mL): <0.1 | 0.1 – 0.5 | 0.5 – 1 | >1

Table S3. IC$_{50}$ and IC$_{80}$ values of BNT351 against the AMP panel, related to Figure 2B

Virus strain	Clade	BNT351 IC$_{50}$	BNT351 IC$_{80}$
H703_0013_090Es	C	0.11	0.46
H703_0015_110s	C	0.58	1.71
H703_0109_210s	C	0.5	1.74
H703_0132_200s_10H3	C	0.21	0.77
H703_0157_090s	C	0.01	0.04
H703_0309_100s	C	0.03	0.15
H703_0322_130s_M1I	C	0.13	0.42
H703_0406_240_RE_e11E4s	C	>10	>10
H703_0406_240_RE_e12B2s	C	2.42	6.91
H703_0455_160_RE_cs	C	>10	>10
H703_0482_250Es	C	0.13	0.32
H703_0514_150Es_A4_V02_028	C	<0.005	0.01
H703_0514_150Es_F4_V03_024	C	1.24	3.91
H703_0514_150s_6C1	C	1.94	5.65
H703_0520_030Es	C	0.02	0.07
H703_0578_130_RE_cs	C	0.06	0.18
H703_0597_190_RE_e5A1s	C	0.03	0.2
H703_0636_200Es	C	0.22	1.15
H703_0739_110s	C	0.01	0.06
H703_0790_190_RE_e5G1s	C	>10	>10
H703_0795_040s	C	3.57	7.67
H703_0805_770_RE_e10C9s	C	0.17	0.93
H703_0805_770_RE_e10F8s	C	2.99	>10
H703_0842_200Es	C	>10	>10
H703_0860_150Es	C	0.01	0.06
H703_0902_140_RE_p01s	C	0.01	0.05
H703_0926_070s_2H2	C	1.7	6.15
H703_0944_180s	C	1.01	2.59
H703_0948_170_RE_e4D2s	C	0.34	1.68
H703_0967_040s	C	0.01	0.06
H703_0993_110_RE_cs	C	0.09	0.26
H703_1026_120Es_A5	C	0.27	0.56
H703_1034_190_RE_e5D5s	C	0.12	0.56
H703_1060_080s	C	0.53	1.9
H703_1194_150_RE_cs	C	0.36	1.84
H703_1357_230Es_H1	C	0.1	0.85

Virus strain	Clade	BNT351 IC$_{50}$	BNT351 IC$_{80}$
H703_1383_240_RE_e5D3s	C	0.01	0.03
H703_1407_090s_4G4	C	0.1	0.3
H703_1446_040s	C	0.02	0.10
H703_1453_240_RE_e9B7s	C	0.39	0.93
H703_1471_190s	C	0.15	0.33
H703_1515_120Es	C	0.13	0.92
H703_1551_143s_2G8	C	0.01	0.07
H703_1670_220_RE_cs	C	1.61	4.96
H703_1675_G613s	C	0.03	0.11
H703_1687_100Es	C	0.03	0.11
H703_1689_110_RE_e4E6s	C	0.06	0.21
H703_1714_080c	C	0.04	0.12
H703_1750_140Es	C	0.21	0.89
H703_1758_260_RE_cs	C	0.07	0.31
H703_1783_170Es	C	0.67	2.71
H703_1789_230_RE_e3A3s	C	0.25	0.61
H703_1798_170_RE_p01s	C	>10	>10
H703_1807_240_RE_cs	C	0.51	3.8
H703_1828_220Es	C	0.03	0.11
H703_1848_190_RE_e6D1s	C	0.1	0.28
H703_1889_100Es	C	0.62	1.01
H703_1945_090s_2A3	C	<0.005	0.03
H703_1945_090s_2F1	C	0.02	0.08
H703_1945_090s_3D5	C	0.02	0.08
H703_2018_240_RE_e6A1s	C	0.08	0.34
H703_2038_260_RE_e5C3s	C	>10	>10
H703_2117_110_RE_e2A10s	C	0.07	0.21
H703_2149_060_RE_eB10s	C	0.01	0.05
H703_2304_150_RE #41	C	0.02	0.14
H703_2539_070_RE_e6F6s	C	0.13	0.49
H703_2631_150_RE_e2F8s	C	0.01	0.08
H703_2769_050_RE_p01s	C	1.41	3.4
H703_2788_030Es_B1	C	0.68	0.97
H703_2934_110_RE_cs	C	0.05	0.2
V703_0015_110_RE_pblib002_s	C	2.38	>10

Virus strain	Clade	BNT351 IC$_{50}$	BNT351 IC$_{80}$
V703_0132_200_RE_pblib002_s	C	0.2	1.22
V703_0309_100_RE_pblib002_s	C	0.05	0.12
V703_0458_260_RE_sga6H5_s	C	0.05	0.48
V703_0510_260_RE_pblib001_s	C	0.44	1.58
V703_0510_260_RE_pblib002_s	C	0.7	2.21
V703_0510_260_RE_pblib003_s	C	0.78	2.44
V703_0514_150_RE_pblib004_s	C	0.65	2.43
V703_0597_190_RE_pblib002_s	C	0.06	0.46
V703_0597_190_RE_pblib003_s	C	0.07	0.27
V703_0712_250_RE_pblib001_s	C	0.25	0.6
V703_0712_250_RE_pblib002_s	C	0.04	0.13
V703_0790_190_RE_pblib002_s	C	>10	>10
V703_1034_190_RE_pblib002_s	C	0.18	0.52
V703_1034_190_RE_pblib003_s	C	0.05	0.24
V703_1104_100_RE_pblib001_s	C	0.15	0.57
V703_1194_150_RE_pblib002_s	C	0.14	1.02
V703_1240_201_RE_pblib003_s	C	0.74	1.8
V703_1240_201_RE_sga3D1_s	C	>10	>10
V703_1240_201_RE_sga4F1_s	C	0.34	1.25
V703_1255_260_RE_pblib003_s	C	>10	>10
V703_1298_080_RE_pblib002_s	C	0.05	0.38
V703_1383_240_RE_pblib002_s	C	0.01	0.04
V703_1383_240_RE_pblib003_s	C	<0.005	0.01
V703_1407_090_RE_pblib003_s	C	0.08	0.24
V703_1453_240_RE_pblib002_s	C	0.13	0.63
V703_1515_120_RE_pblib002_s	C	0.03	0.09
V703_1586_210_RE_pblib002_s	C	2.39	6.16
V703_1586_210_RE_sga3E8_s	C	1.12	3.13
V703_1714_080_RE_pblib002_s	C	0.06	0.16
V703_1855_162_RE_pblib003_s	C	0.09	0.34
V703_1915_250_RE_sgaA3_s	C	0.15	0.34
V703_2141_160_RE_sga3D6_s	C	0.64	1.47
V703_2372_170_RE_con_s	C	0.06	0.23
V703_3000_090_RE_pblib001_s	C	0.01	0.03
V703_3000_090_RE_pblib002_s	C	0.04	0.15
GeoMean		0.13	0.40

Color code (µg/mL):	<0.1	0.1 – 0.5	0.5 – 1	>1

Table S4. Cross-resistance between BNT351 and FDA-approved entry inhibitors, related to Figure 2C

HIV-1 strain	Clade	Tropism	BNT351 (µg/ml)		Maraviroc (µg/ml)		Ibalizumab (µg/ml)		Fostemsavir (nM)	
			IC_{50}	IC_{80}	IC_{50}	IC_{80}	IC_{50}	IC_{80}	IC_{50}	IC_{80}
BZ167.12	B	CXCR4	0.015	0.050	>25	>25	0.055	0.104	0.077	0.217
CNE57	B	CXCR4	0.008	0.024	>25	>25	0.015	0.034	0.426	1.099
HXB2.DG	B	CXCR4	0.007	0.028	>25	>25	>25	>25	0.193	0.678
M02138	CRF01_AE	CXCR4	3.979	>25	>25	>25	0.036	0.109	>500	>500
MN.3	B	CXCR4	0.003	0.015	0.010	>25	>25	>25	1.995	9.29
HIV-89.6	B	Dual Tropic	0.051	0.143	>25	>25	0.094	0.318	0.367	0.988
KER2008.12	A1	Dual Tropic	0.073	0.290	>25	>25	0.059	0.176	>500	>500
P1655_C8_B5_1	F1	Dual Tropic	0.078	0.264	0.0005	0.009	>25	>25	>500	>500
WEAU-d15.410.787	B	Dual Tropic	0.044	0.125	>25	>25	0.039	>25	0.066	0.169
THRO4156.18	B	CCR5	1.286	13.666	0.003	0.020	0.073	0.518	0.302	0.75
T278-50	CRF02_AG	CCR5	>25	>25	0.002	0.009	0.330	>25	0.038	0.106
CNE53	BC	CCR5	0.054	0.265	0.002	0.007	>25	>25	0.083	0.232
Q23.17	A1	CCR5	0.013	0.048	0.003	0.016	>25	>25	2.832	9.52
Q769.d22	A1	CCR5	0.020	0.070	0.001	0.002	>25	>25	20.765	111.566
6041.v3.c23	AC	CCR5	0.014	0.040	0.001	0.002	>25	>25	1.732	4.716
0815.v3.c3	ACD	CCR5	0.014	0.051	<0.0003	0.001	>25	>25	0.379	1.632
BB1006-11.C3.1601	B	CCR5	0.040	0.195	0.002	0.006	>25	>25	0.103	0.266
TRJO4551.58	B	CCR5	0.080	0.208	0.003	0.014	>25	>25	0.158	0.437
001428-2.42	C	CCR5	0.010	0.023	0.002	0.007	>25	>25	10.515	84.717
Ce703010054_2A2	C (T/F)	CCR5	0.038	0.175	<0.0003	0.001	>25	>25	3.601	15.474
CAP45.2.00.G3	C	CCR5	>25	>25	0.001	0.004	0.032	0.053	6.468	34.762
Ce2060_G9	C (T/F)	CCR5	0.222	0.754	0.001	0.004	>25	>25	0.401	1.078
Du172.17	C	CCR5	>25	>25	0.002	0.013	0.033	0.167	1.104	2.995
ZM109F.PB4	C	CCR5	3.480	>25	0.0005	0.002	6.579	>25	0.094	0.261
ZM135M.PL10a	C	CCR5	2.056	14.270	0.0008	0.003	0.058	>25	334.71	>500
3016.v5.c45	D	CCR5	0.744	2.933	<0.0003	<0.0003	0.030	0.084	0.124	0.028
A07412M1.vrc12	D	CCR5	0.056	0.252	0.004	0.017	4.611	>25	1.712	5.837
P1981_C5_3	G	CCR5	7.665	>25	0.004	0.018	0.030	0.289	27.059	125.16
X1632_S2_B10	G	CCR5	0.017	0.078	0.003	0.014	>25	>25	6.36	24.571

µg/ml
**<0.1**
**0.1 – 0.5**
**0.5 – 1**
**>1**

nM
**<0.1**
**0.1 – 1**
**1 – 10**
**>10**

Table S5. Extended pharmacokinetic parameters, related to Figure 4B

Model	Dose (mg/kg)	Animal	C_{max} (µg/mL)	$AUC_{0\text{-}inf}$ (h x mg/mL)	$AUC_{0\text{-}inf}$/Dose (h x kg/mL)	Extrapolated AUC (%)	CL (mL/h/kg)	Vss (mL/kg)	$t_{1/2}$ (d)	MRT (d)
Tg32 SCID mouse	5	13	119.5	11.4	2.3	8.87	0.44	145	11.4	13.8
		14	123.6	12.0	2.4	4.6	0.42	119	7.5	11.9
		15	110.9	16.5	3.3	12	0.3	122	11.4	16.7
		16	124.1	18.1	3.6	8.93	0.28	93	10.1	14.0
	30	17	975.5	145.2	4.8	21.58	0.21	114	19.3	22.9
		18	978.3	119.1	4.0	11.66	0.25	99	12.0	16.4
		19	971.8	106.7	3.6	11.27	0.28	108	11.7	16.0
		20	875.9	154.9	5.2	14.58	0.19	86	13.2	18.6
Cynomolgus macaque	5	1001	133.4	39.8	8	11	0.126	74.0	18.0	24.5
		1002	145.4	37.4	7.5	11.96	0.134	80.4	21.6	25.1
		1003	130.2	30.8	6.2	6.06	0.162	72.1	15.3	18.5
	45	2001	1046.1	336	7.5	10.27	0.134	74.8	21.5	23.3
		2002	1121.9	140.1	3.1	8.04	0.321	46.4	4.0	6.0
		2003	1049.2	274.4	6.1	7.83	0.164	82.9	15.0	21.1

AUC = area under the concentration-time curve; Cmax = maximum serum concentration; CL = clearance; MRT = mean residence time; $t_{1/2}$ = half-life; Vss = steady state volume of distribution
